# Ensemble Inference and Inferability of Gene Regulatory Networks

**DOI:** 10.1371/journal.pone.0103812

**Published:** 2014-08-05

**Authors:** S. M. Minhaz Ud-Dean, Rudiyanto Gunawan

**Affiliations:** Institute for Chemical and Bioengineering, ETH Zurich, Zurich, Switzerland; University of Pittsburgh, United States of America

## Abstract

The inference of gene regulatory network (GRN) from gene expression data is an unsolved problem of great importance. This inference has been stated, though not proven, to be underdetermined implying that there could be many equivalent (indistinguishable) solutions. Motivated by this fundamental limitation, we have developed new framework and algorithm, called TRaCE, for the ensemble inference of GRNs. The ensemble corresponds to the inherent uncertainty associated with discriminating direct and indirect gene regulations from steady-state data of gene knock-out (KO) experiments. We applied TRaCE to analyze the inferability of random GRNs and the GRNs of *E. coli* and yeast from single- and double-gene KO experiments. The results showed that, with the exception of networks with very few edges, GRNs are typically not inferable even when the data are ideal (unbiased and noise-free). Finally, we compared the performance of TRaCE with top performing methods of DREAM4 *in silico* network inference challenge.

## Introduction

The discovery and analysis of biological networks have important applications, from finding treatment of diseases to engineering of microbes for the production of drugs and biofuels [1]–[4]. With continued advances in high throughput and omics technology, the inference of biological networks from omics data has received a great deal of interest. In particular, the inference of gene regulatory networks from gene expression data constitutes a major research topic in systems biology. In the last decade, the number of methodologies that are dedicated for the GRN inference has increased tremendously [5]–[7].

The Dialogue for Reverse Engineering Assessments and Methods (DREAM) project is a community-wide effort initiated to fulfill the need for a rigorous and fair comparison of the strengths and weaknesses of methods for the reverse engineering of biological networks from data. To this end, challenges involving the inference of cellular networks are organized on a yearly basis (http://www.the-dream-project.org/challenges). Specifically, the inference of GRN has become a major focus of several DREAM challenges. The outcomes of these challenges indicate that the state-of-the-art algorithms for GRN inference are unable to provide accurate and reliable network predictions, even when large expression datasets are available and the number of genes is small (10–100 genes) [8]–[10]. Nonetheless, a crowd-sourcing strategy that combines the predictions of different inference methods has been shown to be more reliable than any individual method [10].

Whether or not a direct regulation of one gene by another can be correctly inferred depends not only on the ability of an inference method to extract the relevant information from data, but also on the availability of such information in the data. In general, the information content of data is determined first and foremost by the conditions of the experiments. If the required information is unavailable or incompletely available, then the inference problem is underdetermined. In such a case, the network is not inferable from the data regardless of the method used.

The underdetermined nature is not exclusive to the inference of GRNs. Much of the difficulty in the inverse modeling of signaling and metabolic networks can also be attributed to the lack of inferability or identifiability of model structure and parameters [11]–[14]. As the inference problem is underdetermined, there exist multiple solutions which are indistinguishable. The lack of model identifiability has motivated a paradigm shift toward ensemble modeling [15]–[18]. While such a strategy has begun to gain traction in the modeling of signaling and metabolic networks, the ensemble paradigm has not been widely used in the inference of GRNs. Also, since the network representation and data for GRNs differ markedly from those for signaling and metabolic networks, existing algorithms for ensemble modeling cannot be directly applied for the inference of GRN.

In this work, we introduce new framework and algorithms, called **T**ransitive **R**eduction **a**nd **C**losure **E**nsemble (TRaCE), for the ensemble inference of GRNs. Specifically, TRaCE produces the lower and upper bounds of the ensemble, i.e. the smallest network and the largest network that limit the complexity of networks in the ensemble. As the size of the ensemble reflects the uncertainty about the GRN inference, the bounds can also be used to analyze the inferability of GRNs. In this study, we have used TRaCE in two applications. First, we investigated the inferability of random GRNs and the GRNs of *E. coli* and *S. cereviseae* given steady-state gene expression data of single- and double-gene KO experiments. Then, we applied TRaCE to simulated gene expression data, generated in the same manner as the DREAM 4 *in silico* network inference challenge, and compared the performance of TRaCE with existing methods.

## Methods

### Theoretical Foundation

#### Definitions

Here, we provide a short synopsis of basic concepts in graph theory that are necessary for the development of our algorithm. A *graph* 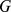 is an ordered pair 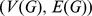, where 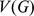 is the set of vertices (or nodes) and 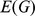 is the set of edges. The number of vertices 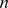 and the number of edges 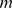 are called the order and the size of the graph, respectively. An edge 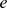 is defined by the pair 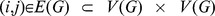, describing the existence of a relationship between two vertices 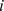 and 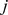. In this case, the edge 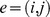 is said to be *incident* to the vertices 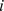 and 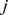. The set of edges of a graph 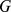 that are incident to a vertex 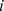 is denoted by 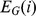, while the cardinality of 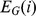 is called the degree of the vertex 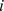. Similarly, the set of edges that are incident to a set of vertices 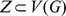 is denoted by 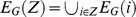. A graph 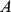 is a *subgraph* of 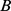, denoted by 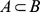, if 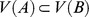 and 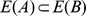. In this case, 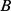 is called the *supergraph* of 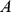, and is also said to contain 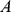. Furthermore, the union of two graphs 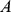 and 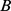 is denoted by 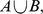 where 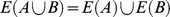 and 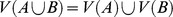. The intersection of two graphs 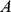 and 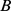 is denoted by 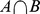, where 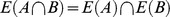 and 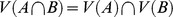. Finally, the difference between two graphs 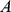 and 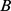 with 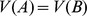 is denoted by 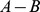 and defined as the set of edges in the graph 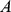 that do not belong to the graph 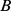, i.e. edges in the set difference 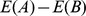.

A *directed edge* is an ordered pair 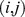, representing an edge from the vertex 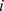 pointing to the vertex 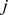. A *directed graph* or *digraph* 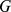 is a graph in which all of its edges are directed. A *directed path* is a sequence of vertices such that there exists a directed edge from one vertex to the next vertex in the graph. The first vertex in a directed path is called the *start vertex*, and the last is called the *end vertex*. A *directed cycle* is a directed path where the start and the end vertices are the same. A *directed acyclic graph* (DAG) is a digraph which does not contain any cycle. The *adjacency matrix* of a digraph 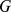 of order 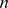, denoted by 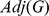, is an 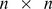 matrix with 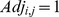 when 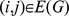, and 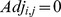 otherwise. In other words, the non-zero elements of the adjacency matrix represent all directed edges from any node 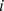 to another node 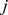 in the graph 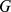. Meanwhile, the *accessibility matrix* of 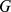, denoted by 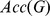, is an 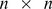 matrix with 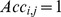 when there exists a directed path from node 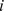 to node 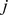, and 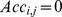 otherwise. When 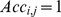, vertex 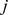 is said to be *accessible* from the vertex 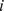.

A strongly connected component or strong component of a digraph 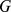 is a maximal subset of nodes in 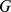 where any two nodes in the subset are mutually accessible. Every pair of nodes that are part of a directed cycle belong to the same strong component, while any node that is not part of a cycle is a strong component of its own. The *condensation* of a digraph 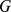 is the DAG of the strong components of 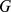, which is generated by lumping the nodes belonging to a cycle into a single node and replicating the edges that are incident to any of these nodes onto the lumped node [19].

**Figure 1:**
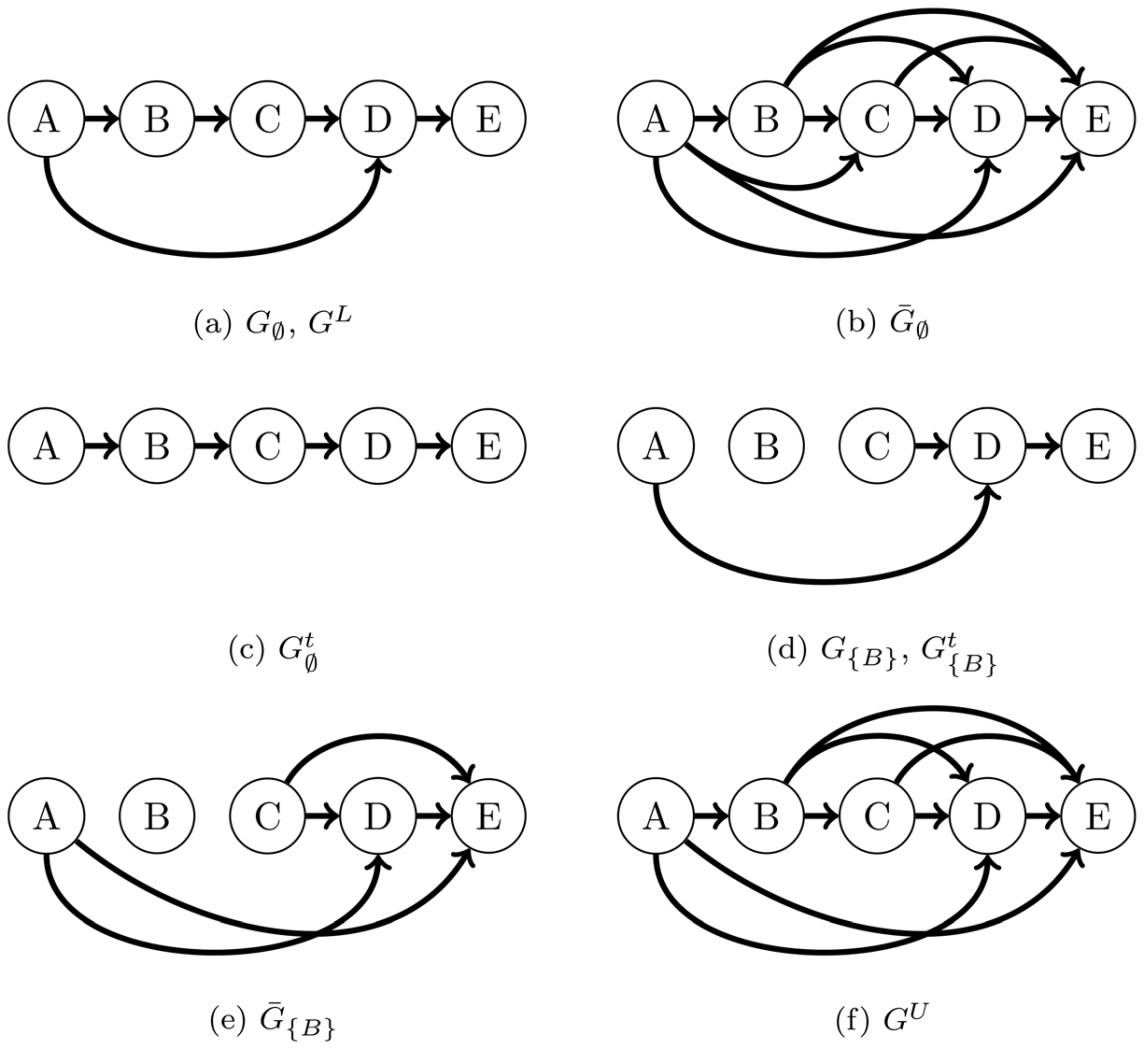
(a) An example of a directed graph 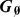. (b) The transitive closure 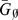 (in this case, 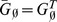 since 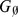 is a DAG). (c) The transitive reduction 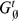 of 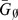. (d) The directed graph 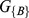 associated with 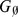. (e) The transitive closure 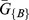. In this case, the transitive reduction 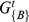 happens to be the graph 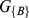. (f) The ensemble upper bound 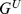 obtained from 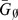 and 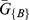. The ensemble lower bound 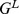 obtained from 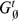 and 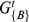 happens to be the graph 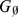.

A digraph is *transitive* if for every pair of vertices 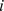 and 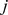, there exists an edge 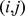 when there is a directed path from 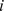 to 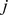. The *transitive closure* of a digraph 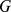, denoted by 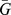, is the smallest transitive supergraph of 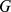 (with the fewest edges) [20]. When 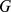 is a DAG, we denote the transitive closure of 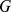 as 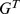. As shown in Fig. 1(a)-(b), the transitive closure of a digraph can be generated by adding a directed edge 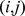, whenever a directed path exists from vertex 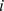 to vertex 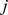. Note that the accessibility matrix of a digraph is the adjacency matrix of its transitive closure, i.e. 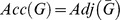. For a digraph 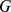, the set of digraphs that have the same transitive closure 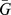 is denoted by 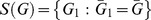. The *transitive reduction* of 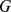, denoted by 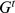, is defined as the smallest member of 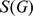 in size (i.e. the graph with the fewest edges). The transitive reduction of a DAG is unique, given by 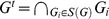 [20]. An algorithm for obtaining transitive reduction has been previously developed [19], in which any directed edge 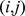 is pruned whenever there exists a directed path from 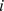 to 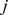 that does not include 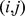 (for example, see Fig. 1(c)). Note that the transitive reduction of a digraph with cycles is not unique.

#### Inference of Network Ensemble Bounds

In this work, we consider the inference of GRNs as digraphs, where the nodes correspond to the genes and the directed edges represent the gene regulatory interactions. An edge 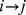 implies that the expression of gene 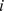 influences the expression of gene 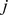. In the following, the GRN of interest is denoted by the digraph 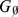. For any set of genes 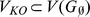, we use 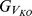 to denote a subgraph of 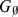 that results from removing all edges incident to the genes in the set 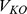, 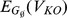. In other words, 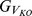 is the digraph with 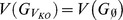 and 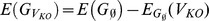. For example, 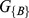 associated with 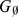 in Fig. 1(a) is the graph with all edges incident to gene 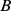 removed, as shown in Fig. 1(d). Here, we interchangeably use the notations for a graph 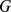 and its adjacency matrix 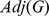.

Gene KO experiments are commonly performed for the purpose of GRN inference. In these experiments, the resulting data typically consist of gene expression profiles taken after the effects of the gene perturbation have reached steady state. While temporal gene expressions are increasingly measured, here we focus on using more commonly available steady-state expression data. The treatments of time-series measurements and observational data are left to future publications. Many network inference algorithms have been developed for using data of gene KOs [7], [9], and most of these algorithms produce a single network prediction. In contrast, an ensemble inference strategy is adopted in this work.

In order to illustrate the limitation of using steady-state gene expression data for GRN inference, we consider a GRN 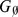 described by the graph in Fig. 1(a). Here, KO of gene 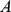 is expected to cause changes in the expression of genes 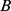, 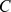, 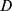 and 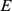 at steady state, even though 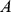 directly regulates only 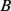 and 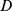. This simple illustration demonstrates that we cannot in principle discriminate direct and indirect gene regulations from steady-state gene KO expression data [19]. In general, genes that are differentially expressed upon knocking out gene 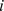 in the GRN correspond to those that are directly and indirectly regulated by gene 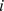, i.e. vertices in 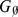 that are accessible from the vertex 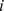. Motivated by such a limitation, in TRaCE we first convert gene KO data into gene accessibility lists or matrices. As the minimum input, TRaCE requires the complete dataset of single-gene KO experiments, from which one can construct the accessibility matrix 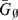. More specifically, the 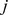-th element in the 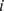-th row of 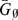 (i.e. 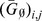) is set to 1 when gene 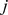 is differentially expressed in the KO experiment of gene 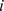. The other elements of 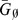 are set to 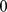. The detailed procedure of differential expression analysis adopted in this work is described in the Numerical Implementation section.

For data of multi-gene KO experiments, we consider the accessibility matrix of 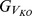 for an appropriately chosen set of genes 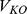. In principle, we can determine 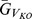 from the complete set of experiments involving KOs of the genes in the set 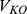 and an additional gene 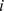 for all 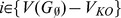. These experiments are equivalent to performing single-gene KOs of the GRN 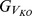, and therefore 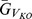 can be obtained by following the same procedure as that for 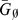 above. As an illustration, consider the GRN in Fig. 1(a) with 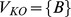. The graph 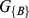 is given in Fig. 1(d). In this case, we can construct the accessibility matrix 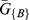 from the data of two-gene KO experiments, namely 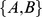, 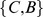, 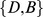 and 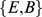 KOs. As these experiments differ from each other in only one gene while sharing the KO of gene 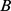, the differential expression analysis of the data thus correspond to changes in the expression of the GRN 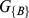 caused by a single-gene KO. Consequently, in this analysis, genes that are found to be differentially expressed in the KO of 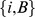 are those that are accessible from gene 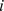 (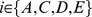) in the graph 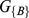. For example, the KO of 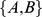 is expected to cause differential expression in genes 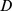 and 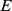. The full accessibility matrix of 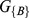 is illustrated by the digraph in Fig. 1(e).

We can generalize the simple example above to any set of genes 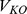 that could be derived from the available multi-gene KO experiments. More specifically, we set 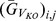 to 1 when knocking-out 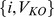 leads to a differential expression of gene 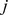 with respect to its expression level in 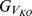. The remaining elements of 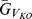 are set to 0. Unfortunately, the construction of 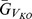 of a large GRN 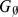 would proportionally require a high number of KO experiments (the number of KO experiments is 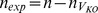, where 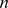 and 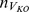 is the number of genes in 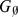 and 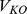, respectively). However, when 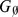 is sparse, 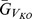 differs from 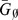 for only a few elements and importantly, these elements can be determined from 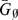 (see the next section).

In the theoretical development below, we assume that the accessibility matrices 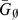 and 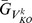 for 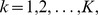 have already been obtained from the expression data. Here, 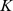 denotes the total number of accessibility matrices involving subgraphs of the GRN 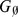 that can be constructed from data. For example, from the dataset of the complete double-gene KO experiments, we can obtain the accessibility matrix 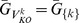 for 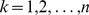 (here, 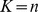). In TRaCE, we consider the ensemble containing all digraphs that are consistent with the accessibility matrices 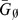 and 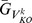
's, which is the set:
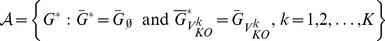
(1)where 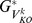 is the digraph with 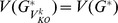 and 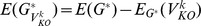. Note that the GRN 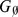 is a member of the ensemble 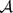. The size of the ensemble is a direct measure of uncertainty in the network inference problem. A GRN is therefore deemed inferable when the ensemble only contains a single (unique) network.

As the number of digraphs in the ensemble is often very large, in TRaCE we generate only the lower and upper bounds of the ensemble, denoted by 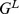 and 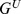, respectively. The bounds are defined such that each digraph in the ensemble is a supergraph of 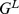 and a subgraph of 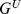. For GRNs without any cycle (i.e. DAGs), the lower and upper bound GRNs can be obtained from the accessibility matrices of 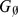 and 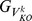
's (i.e. 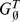 and 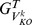
's) and their transitive reductions (i.e. 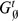 and 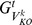
's), using the following equations (for details see the Numerical Implementation section):
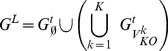
(2)

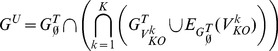
(3)where 
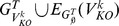
 denotes the digraph with vertices 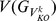 and edges 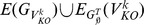. Without any 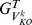, the upper bound of the ensemble is simply given by the accessibility matrix 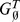 and the lower bound is the transitive reduction 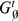. As 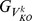 is a subgraph of 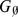, the transitive closure 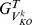 is also a subgraph of 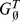. In Eq. (3), the upper bound is constructed starting from 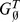 in which edges are removed based on 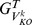. Here, edges incident to 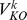 are not altered during the intersection of the accessibility matrix 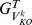. For example, consider again the GRN in Fig. 1(a) with the accessibility matrices 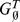 and 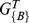 in Figs. 1(b) and 1(e). The resulting upper bound 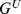 from the combination of these accessibility matrices will have one fewer edge than 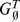, which is the edge 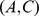 (see Fig. 1(f)). Thus, the size of the upper bound is generally reduced with the incorporation of 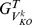
's. On the other hand, according to Eq. (2), the lower bound becomes larger with the inclusion of every available 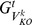. In the same example above, the transitive reduction of 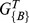 happens to be the graph 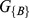 (i.e. in this case 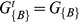). Here, the combination of 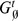 and 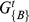 in Figs. 1(c) and 1(d), respectively, gives the lower bound 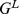 that is equal to 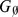. However, in general, 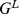 is a subgraph of 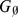.

Theorem 1 establishes 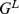 and 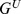 in Eqs. (2)-(3) as valid lower and upper bounds of the set 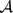 for DAGs.

**Theorem 1:** For 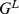 and 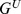 described in Eqs. (2)-(3), the following relationship applies:
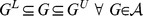



*Proof of* 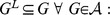 For any edge 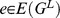, Eq. (2) implies that either 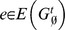 or 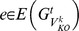. Therefore, we have either:

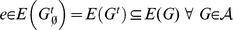
, or

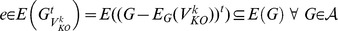
. 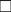

*Proof of* 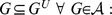 If 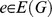 for some 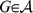, then 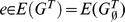. In addition, this edge satisfies either: 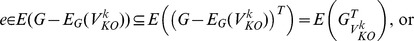


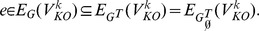



Therefore, 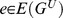. 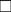


**Remark**: Since 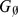 is a member of 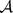, 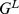 and 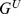 can also be thought as the lower and upper bounds of 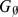, i.e. 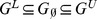. For DAGs, the members of the set 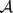 can be obtained by combinatorially adding edges in the set 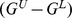 to 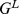. Therefore, the dimension of 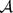 is equal to 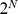 where 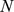 is the difference between the number of edges in 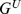 and 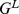. Finally, Theorem 1 guarantees that when 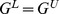, 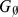 is fully identifiable, i.e. 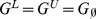.

For digraphs with cycles, the upper bound can still be constructed using Eq. 3 with 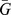 replacing 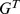. In this more general case, the relationship 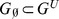 in Theorem 1 is still valid. However, as mentioned earlier, the transitive reduction of digraphs with cycles is not unique. In a previous publication [19], Wagner proposed a procedure in which digraphs are first condensed into DAGs before constructing the transitive reduction [19]. Similarly, in TRaCE, each input accessibility matrix is first condensed and the transitive reduction algorithm is subsequently applied to the DAG of the strong components. Here, edges incident to the condensations of cycles are also removed. Afterwards, the transitive reduction graph is expanded, reversing the condensation step. Except for cycles involving two nodes, edges of any directed cycle cannot be uniquely prescribed and are therefore pruned. The above procedure for reducing digraphs with cycles is referred to as **Con**densation, **T**ransitive **R**eduction and **Ex**pansion (ConTREx). The ConTREx of an accessibility matrix 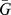, denoted by 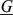, may no longer be a valid transitive reduction (i.e. 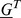 may not necessarily be equal to 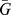). Nonetheless, the lower bound constructed using Eq. (2) with 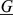
's replacing 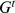
's, satisfies 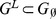. The proof of this relationship is analogous to the one presented for Theorem 1. However, 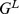 may not be a member of 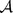 (see Fig. S1). Finally, the enumeration of digraphs with cycles from 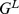 and 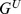 is more complicated than that for DAGs. The main difference is in the generation of all possible cycles among nodes belonging to a particular strong component, constrained by 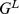 and 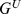 (see an example in Fig. S2).

#### Error Correction and Filter

In practice, the accessibility matrices constructed from data contain errors. Some elements of the accessibility matrices maybe identified as 1 when they should be 0 (i.e. false positive, FP), and some maybe identified as 0 when they should be 1 (i.e. false negative, FN). These errors can affect the lower and upper bound constructed by Eqs. (2)-(3). We denote the erroneous lower and upper bound by 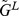 and 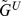, respectively. In this case, neither 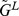 is guaranteed to be a subgraph of 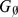, nor 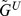 a supergraph of 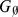 and 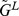.

There are several types of errors affecting 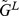 and 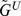. In the first case (Type A error), an edge which is not present in 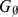 (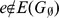) erroneously appears in 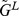 and 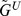 (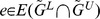). Or, an edge in 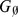 (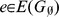) is missing from both 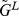 and 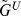 (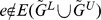). As such error affects both 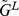 and 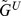 in the same manner, this error is not detectable from 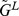 and 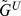. The second case (Type B error) involves either a FP in the accessibility matrix or a FN in the ConTREx matrix. In this case, the resulting bounds are still consistent with each other and are still valid for 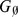. However, the ensemble size and the network uncertainty increase due to this error. In the third case (Type C error), an edge erroneously appears only in 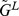, or vice versa, an edge is errorneously missing only from 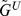 (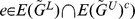, where 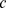 denote the complement of a set). Here, the bounds become inconsistent with each other (i.e., 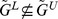). Thus, we refer such errors as *inconsistent edges*, which can be identified by searching for edges belonging to 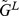 that are not in 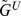 (i.e., from 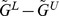). Table 1 illustrates the three types of errors mentioned above.

**Table 1:** Types of Errors in 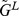 and 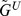.

	Error			
	Type A		Type B	Type C
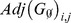	0	1	0 or 1	0 or 1
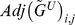	1	0	1	0
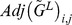	1	0	0	1

A closer scrutiny of Eqs. (2)-(3) reveals that errors from the input accessibility matrices are passed on and compounded in the bounds. For example, FN errors in 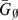 or any 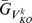 will end up in 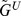, while FPs in 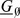 and any 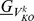 will also appear in 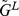. In order to reduce the transmission of errors, we have developed a filter such that only a subset of edges of 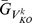 are used for the construction of 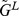 and 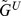. The filter is based on the concept of *testable edges*. Specifically, the testable edges of 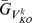 are any edge 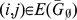 with 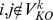, such that there exists a directed path from 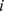 to 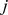 involving one or more genes in 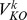. As directed paths involving genes in 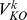 are disconnected in 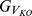, we can potentially verify the existence of the testable edges from 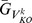 and 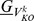. For example, the existence of the edge 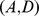 in Fig. 1(a) can be verified using the transitive reduction of 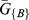, which is the graph shown in Fig. 1(d). Meanwhile, we can establish the absence of the edge 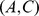 in 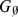 using the accessibility matrix 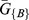 (see Fig. 1(e)). The number of testable edges can also be used to estimate the information content of a 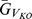, where a higher number of testable edges indicates more informative 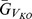.

For a given 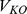, it is straightforward to show that the testable edges are the non-zero entries of the testability matrix:
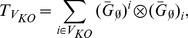
(4)where 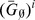 and 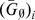 are the *i*-th column and *i*-th row of 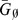, respectively, and 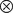 denotes the outer product. During the construction of the lower and upper bound GRNs, the incorporation of each 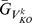 will only need to be performed for the associated testable edges, i.e. non-zero elements of 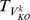. Moreover, as the number of testable edges corresponding to 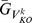 is typically small and as such edges can be determined from 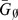, only a few rows of 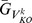 need to be determined from data, i.e. rows of 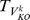 with non-zero entries. Thus, the number of KO experiments for constructing 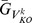 could be relaxed by considering only testable edges.

### Numerical Implementation

The pseudo-codes and the MATLAB implementations of TRaCE are provided in the supporting material and the following website (http://www.cabsel.ethz.ch/tools/trace). Given steady-state gene expression data, we first group the data into datasets according to the KO experiments required for the construction of the accessibility matrices. We perform differential expression analysis for each dataset using Z-score transformation and obtain the corresponding accessibility matrix. We provide two implementations of TRaCE, one with and another without error correction. TRaCE without error correction should only be applied when the input accessibility matrices are error free (e.g. for inferability analysis). In any other scenario, TRaCE with error correction should be used. If desired, a ranked list of gene regulatory predictions can also be generated using the lower and upper bounds of the ensemble and the differential expression analysis.

#### Constructing Accessibility Matrices from Expression Data

**Figure 2:**
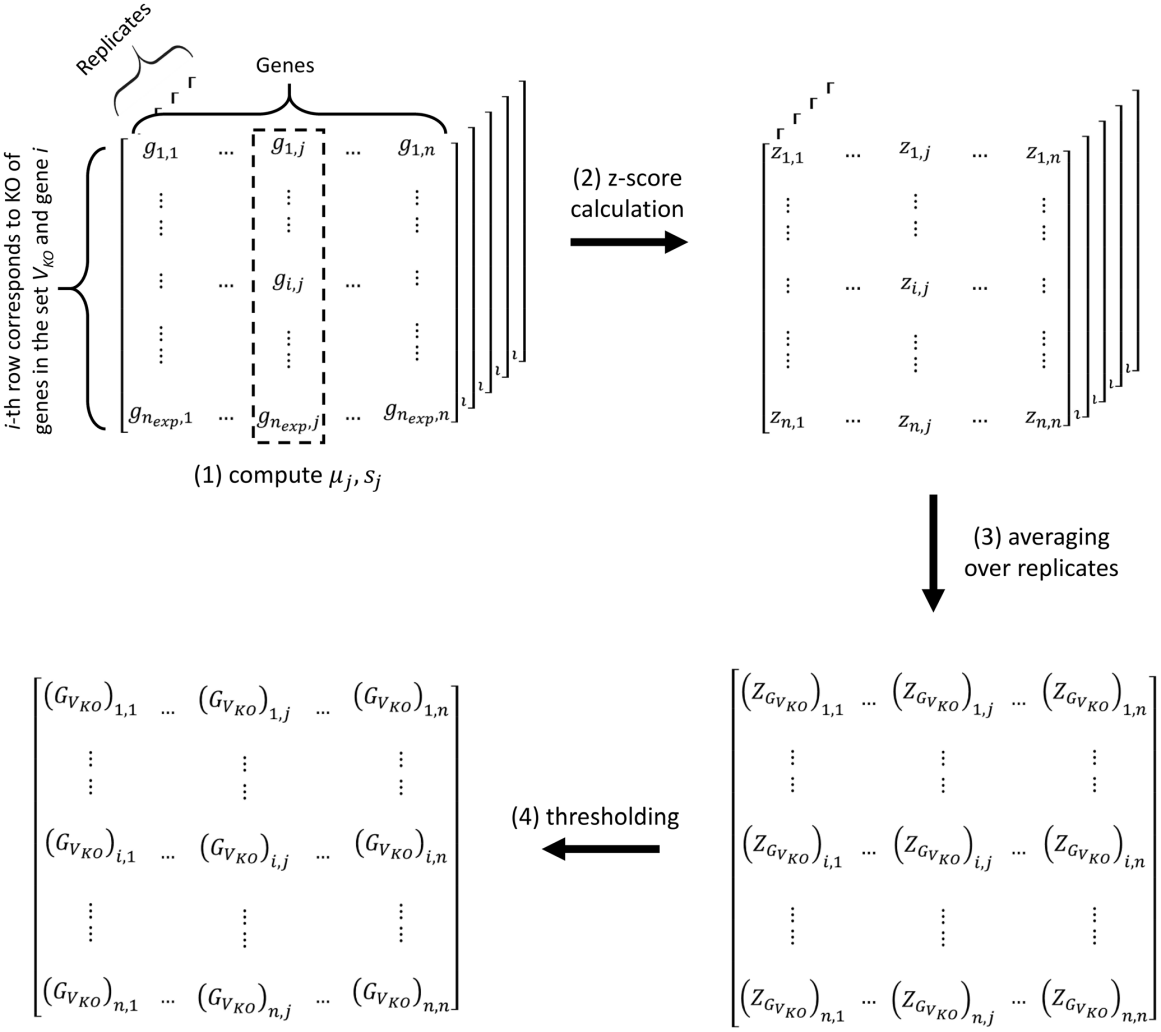
Construction of accessibility matrix 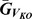 from expression data. The data come from KOs of genes in the set 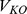 and an additional gene 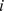, 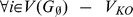. For each replicate, the expression data are arranged into a matrix where the rows correspond to the experiments and the columns correspond to the genes. (1) The sample mean and standard deviation of the expression of gene 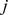, denoted by 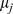 and 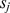, respectively, are obtained using the expression data in the 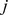-th column of the data matrix. (2) For each replicate, a z-score matrix is computed according to Eq. (5). (3) Subsequently, the z-score matrices are averaged over the technical replicates to give 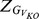. (4) The accessibility matrix 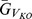 is determined from 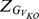 based on a threshold criterion in Eq. (6).

In the case studies, we have employed the Z-score transformation for differential gene expression analysis [21]. Without loss of generality, we describe below the procedure for constructing 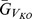 from the complete set of single-gene KOs of 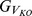, i.e. all possible combinations of 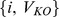 genes KOs. The gene expression dataset is organized into a matrix in which the rows correspond to the experiments and the columns correspond to the genes. Technical replicates are arranged into separate data matrices. For microarray data, the gene expression is typically represented by log-10 transformed fluorescence intensity data. The following procedure is also illustrated in Fig. 2.

1. We first obtain the sample mean 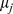 and standard deviation 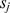 of the expression of each gene 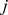 in the dataset. More specifically, for each technical replicate, we calculate the sample mean and standard deviation of the 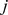-th column in the data matrix. Then, we identify expressions that differ from the mean by more than a specified multiple 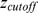 of the standard deviation. We subsequently recompute the sample mean and standard deviation 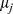 and 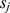 by excluding the data beyond 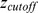. When available, we also use the expression data from the KO experiment of genes 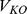 in calculating 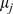 and 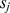.
2. For each replicate, we compute a z-score matrix 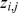 for 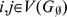 according to [21]

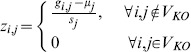
(5)where 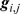 is the expression level of gene 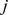 associated with knocking out gene 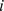 and genes in 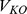. These z-scores reflect the significance of changes in the gene expression with respect to the GRN 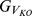.
3. Subsequently, we average the z-score matrices over the technical replicates, producing the overall z-score matrix 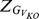.
4. We determine the accessibility matrix 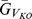 from 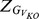 using a threshold, as follows:
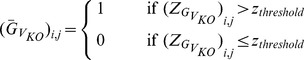
(6)

In our experience, 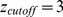 and 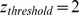 provide reliable network ensembles. In general, choosing higher 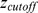 and 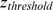 will lead to fewer FPs but more FNs in the accessibility matrix. For the GRN examples considered in this work, the performance of TRaCE does not vary considerably within the selected ranges of 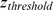 between 1.5 and 2.5 and 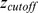 between 2 and 3 (see Results).

#### TRaCE without error correction

**Figure 3:**
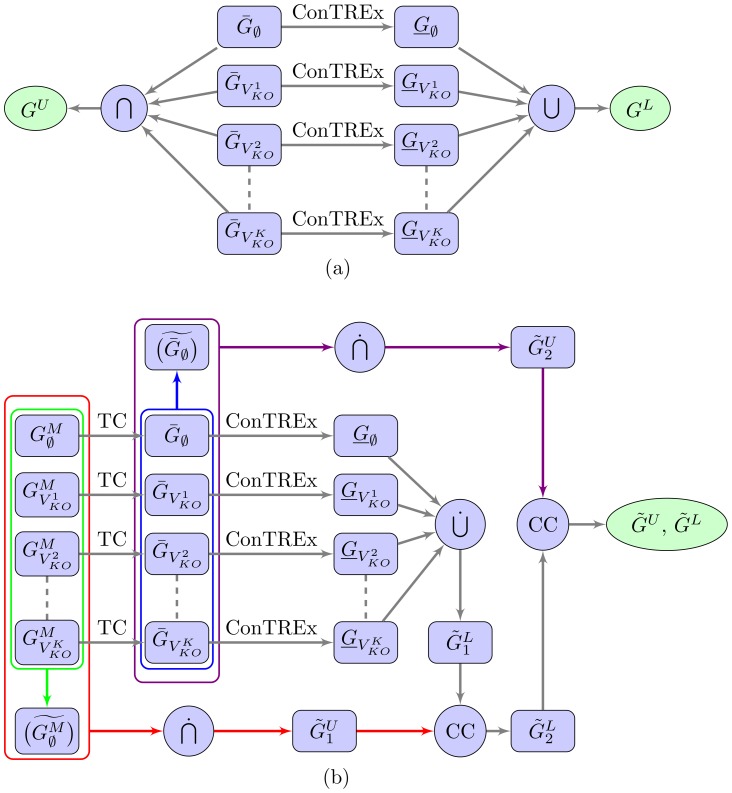
Schematic diagrams of TRaCE with and without error correction. (a) Construction of the lower bound 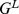 and upper bound 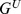 from 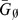 and 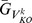
's using TRaCE without error correction. Expression data from gene KO experiments are first converted into accessibility matrices. ConTREx is then applied to each accessibility matrix, removing feed-forward edges and edges incident to vertices belonging cycles with more than 2 nodes. The upper bound is constructed by taking the intersection of the accessibility matrices, while the lower bound is constructed by taking the union of the ConTREx outputs. (b) Construction of the lower bound 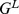 and upper bound 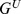 from 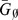 and 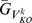
's using TRaCE with error correction. Expression data from gene KO experiments are converted into accessibility matrices 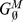 and 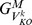
's, where the superscript 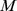 indicates that these matrices may not be transitive due to noise in the measured gene expression levels. Subsequently, the transitive closures of 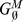 and 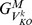
's are created, denoted respectively by 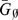 and 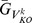
's, and the ConTREx of these closures are evaluated. TRaCE with error correction begins with the preprocessing of 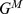
's and 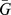
's to produce the corrected matrices 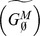 and 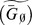, which are required to determine testable edges. For the construction of the lower and upper bounds, the union and intersection of matrices are performed with filtering, denoted by 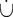 and 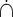, respectively, where only the relevant testable edges are updated. Two candidate upper bounds are obtained, the first from the matrices 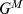
's, denoted by 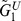, and the second from the matrices 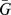
's, denoted by 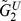. Meanwhile, the initial lower bound estimate, denoted by 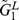, is obtained from the ConTREx matrices. The consistency check (CC) is first applied to the pair 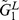 and 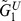 to produce the corrected lower bound 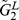, and then to the pair 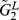 and 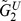 to produce the final estimates of the bounds 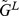 and 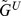. More detailed descriptions of the filtering and consistency check can be found in supporting material (text S1 and text S2).

TRaCE without error correction is implemented as matrix-operations of Eqs. (2) and (3). Briefly, the upper bound 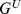 is constructed by performing Hadamard (element wise) multiplications of the accessibility matrices, excluding the rows and columns corresponding to genes in 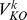. On the other hand, the transitive reduction is based on the algorithm by Wagner [19], which has been re-implemented using matrix operations. When there is no cycle in 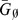 and 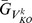
's, the transitive reduction algorithm is applied to each accessibility matrix and the construction of 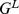 is done by binary additions of the transitive reductions, following Eq. (2). Cycles and genes involved in cycles can be detected from entries of 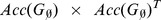 [22]. For GRNs with cycles, the ConTREx procedure is applied to each available accessibility matrix, and the resulting 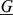 matrices are again combined using binary additions to produce 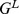. The schematic diagram of the error-free implementation is shown in Fig. 3(a).

#### TRaCE with error correction

The procedure for TRaCE with error correction is illustrated in Fig. 3(b). There are two main steps in this procedure: (1) the construction of lower and upper bounds with filtering and (2) the correction of inconsistent edges. The first main step refers to an implementation of Eqs. (2) and (3) in which the intersection and union operations involving 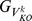 are performed only for testable edges associated with non-zero entries of 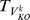. As testable edges are determined from 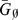, a pre-processing step is performed to reduce errors in 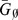. The premise behind the pre-processing step is that an error unlikely affects the same edge, and that testable edges of any 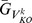 constitute only a small subset of edges in 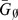 (i.e. the network is sparse). Following this premise, edges that appear in a majority of the accessibility matrices (above a certain threshold) are kept, but are otherwise removed. In our experience, a threshold of 65% gives a good and reliable performance, but any value between 50% to 80% works quite well in the case studies (see Results). A more detailed description of the pre-processing method can be found in text S1, while the filtering algorithm is provided in text S2.

The schematic diagram of TRaCE with error correction is given in Fig. 3(b). We consider two sets of accessibility matrices; the first set comes from differential expression analysis (based on 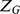
's) and the second set comes from the transitive closure of the first set. We create the second set of matrices since the accessibility matrices identified from differential expressions may not satisfy the transitivity condition due to errors. The pre-processing step above is applied to both sets of matrices. Subsequently, two candidate upper bounds are generated using TRaCE with filtering. The upper bound obtained from the first set of accessibility matrices, denoted by 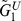, is expectedly smaller (in size) than the bound from the second set, denoted by 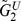. Note that ConTREx is only applicable to transitive digraphs, and therefore is applied only to the transitive closures (i.e. the second set). Using TRaCE with filtering, a candidate lower bound, denoted by 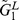, is generated from the results of ConTREx.

The last step in the procedure is to correct inconsistent edges, which is done by voting. For each inconsistent edge, we compared the number of times that the edge is present in the accessibility matrices and the ConTREx results (supporting the presence of the edge), with the number of times that the edge is absent from the accessibility and ConTREx matrices (supporting the absence of the edge). The upper bound is corrected (by addition of this edge) when the presence of the edge receives a (simple) majority vote. Vice versa, the lower bound is corrected (by removal of this edge) when the absence of the edge receives a majority vote. In the case of no majority vote, the edge is added to the upper bound and removed from the lower bound. The detail of the consistency check is described in text S3. As shown in Fig. 3(b), the consistency check and correction are first performed for the pair 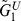 and 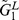, and subsequently the corrected lower bound, denoted by 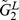, is compared with 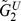 to obtain the final corrected 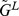 and 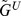.

#### Ranking of Edges from Ensemble Bounds

If desired, a ranked list of edges can be generated using the lower and upper bounds of TRaCE in conjunction with the average z-scores for 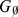, i.e. 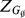. Here, we carry out the ranking of regulatory edges in two phases. In the first phase, we rank subsets of edges according to the lower and upper bounds in the following order: edges in 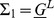, edges in 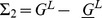, edges in 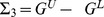, edges in 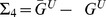, and finally edges in 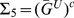. In the second phase, we rank the edges within individual subsets according to the average z-scores. We implement the second phase by first computing the overall scores 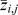 according to
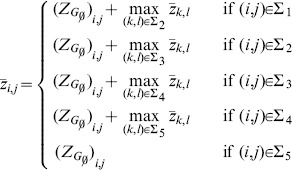
(7)


Following the submission requirement of DREAM 4 network inference challenge, we then assign a confidence score 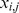 to the edge 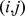 according to:
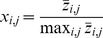
(8)


A score 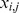 of 1 reflects the highest confidence of the existence of an edge 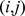, and vice versa a zero confidence score indicates certainty in the inexistence of 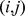. Finally, the ranked list of edges is generated by sorting the edges in decreasing order of confidence scores. A similar procedure, called down-ranking, has been presented in Pinna *et al*. [23], where feed-forward edges are ranked lower than edges in the transitive reduction of the accessibility matrix. However, the down-ranking algorithm is described only for data of single-gene KO experiments.

## Results

### Inferability Analysis

We first applied TRaCE to error-free accessibility matrices of 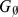 and 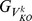 by assuming ideal data (unbiased and error free) for the purpose of inferability analysis. Such an analysis is analogous to *a priori* identifiability analysis in the kinetic modeling of biological networks [12]. Here, we evaluated the network distances between the lower and upper bounds and the GRN, i.e. the numbers of edges in the set 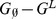 and 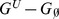, respectively.

#### Random GRNs

We investigated the inferability of random GRNs of orders 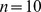 and 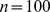 genes. We set the network size (i.e. number of edges) between 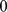 and 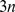 randomly with equal probability, and assigned the edges without any preference. The upper size limit of 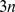 was chosen based on the ratio between the number of edges and the number of nodes in *E. coli* and yeast GRNs [24]. For each random network, we generated 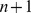 accessibility matrices associated with 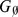 and 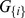 for every 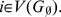 These accessibility matrices correspond to performing the full set of single- and double-gene KO experiments.

**Figure 4:**
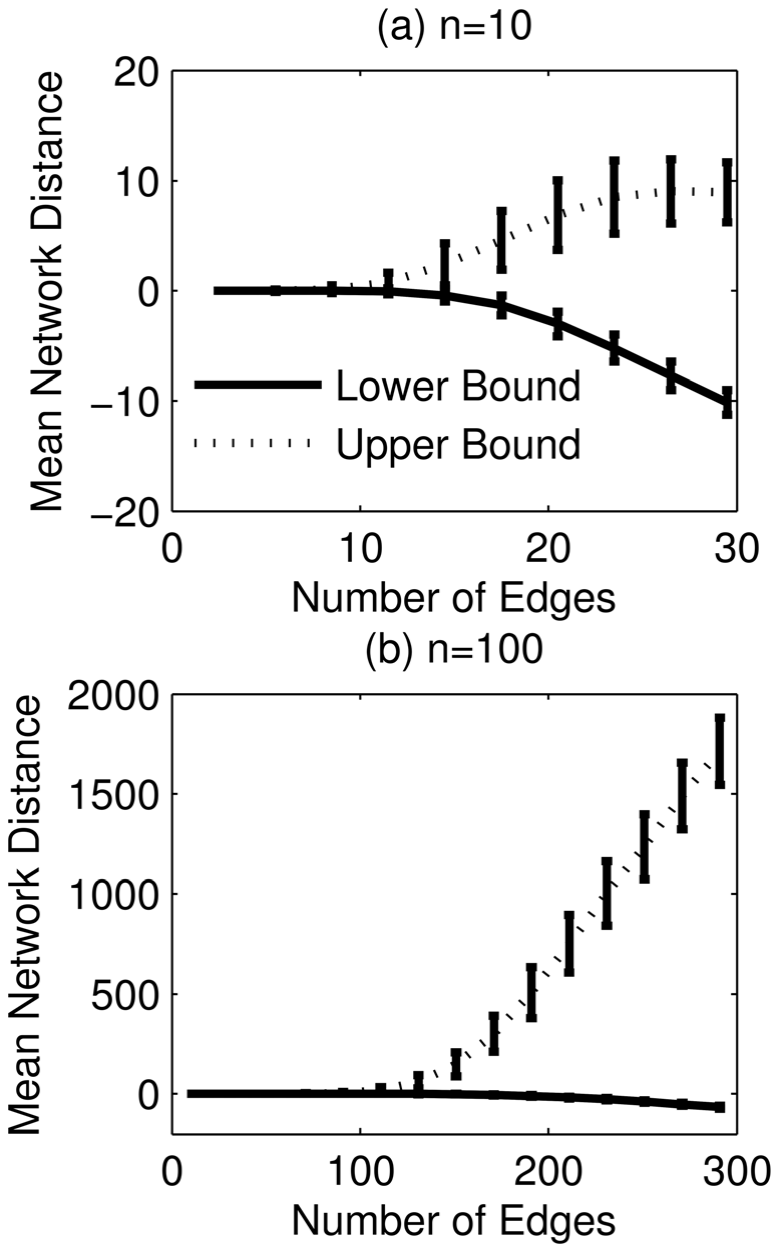
Ensemble inference and inferability of 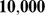 random networks of order (a) 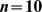 and (b) 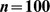 genes. The mean network distances of the lower and upper bounds from 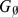 are shown as a function of network size (i.e. number of edges). The error bars indicate the standard deviations.

We applied TRaCE without error correction to construct the ensemble lower and upper bounds for each random network using the aforementioned accessibility matrices. The mean network distances of the bounds from 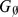 are shown in Figs. 4(a) and (b) as a function of network size. Here, we plotted the network distances of the lower bound using negative numbers and those of the upper bound using positive numbers. By doing so, we could illustrate the distance between the lower and upper bounds in the same plot. In particular, the number of edges in the set 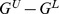 is equal to the distance between the two network distance curves in Fig. 4. Not surprisingly, the network distance increased with the size of the networks, i.e. larger networks are more difficult to infer than smaller networks. The difference between the lower and upper bounds also broadened with network size, indicating higher network uncertainty in the inference of larger GRNs. For networks containing fewer edges than nodes, the GRN 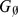 could generally be recovered from 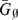 and 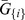
's. Nevertheless, Fig. 4 demonstrated that the GRNs were typically (64% for 10 gene networks and 76% for 100 gene networks) not inferable, since the lower and upper bounds did not converge.

**Figure 5:**
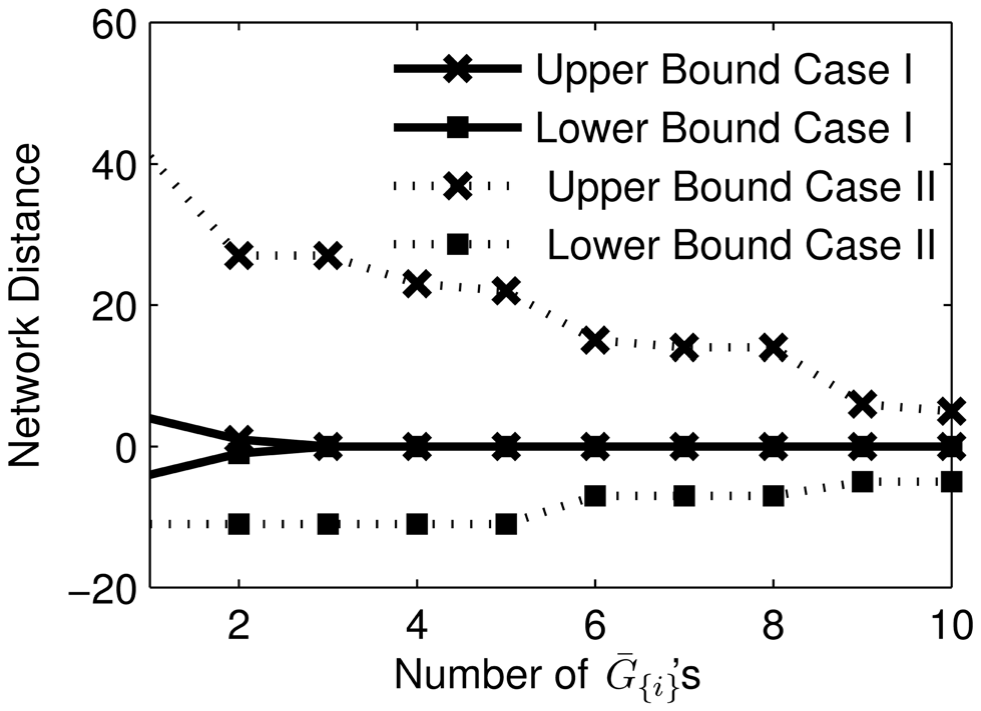
Examples of the ensemble inference of random networks with 10 genes. In case I, the GRN has 8 edges and is inferable from the accessibility matrices 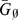 and as few as three 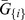
's. In case II, the GRN has 13 edges and is not inferable.

Fig. 5 shows two examples of GRN inference of order 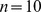 genes. In the first case (case I, 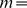 8 edges), 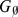 could be recovered from 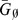 and as few as 3 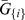
's, while in the second case (case II, 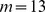 edges), the inference problem was underdetermined. Moreover, the results suggested that 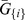
's were not equally informative, as the reduction in the distance between the lower and upper bounds by incorporating an additional 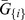 was not uniform.

#### Random scale-free GRNs

Many cellular networks have been shown to be scale-free with a power-law degree distribution [25], where the majority of the nodes have low degrees (1 to 2) and a few nodes (called hubs) are of high degrees. We also tested the performance of TRaCE using random scale-free networks. Here, we constructed two sets of 5000 scale-free GRNs with order 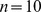 and 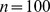 genes using the Barabási–Albert model [26]. Briefly, the GRNs were grown from a random seed network of small size (with 3 vertices) by sequentially adding nodes to the network. For each node addition, between 1 and 5 new edges were inserted to the network connecting the new node with existing ones, in a manner such that the degree distribution decayed exponentially. Again, for the purpose of inferabiliity analysis, we generated 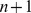 error-free accessibility matrices 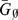 and all 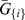
's, equivalent to having ideal data from single- and double-gene KO experiments.

**Figure 6:**
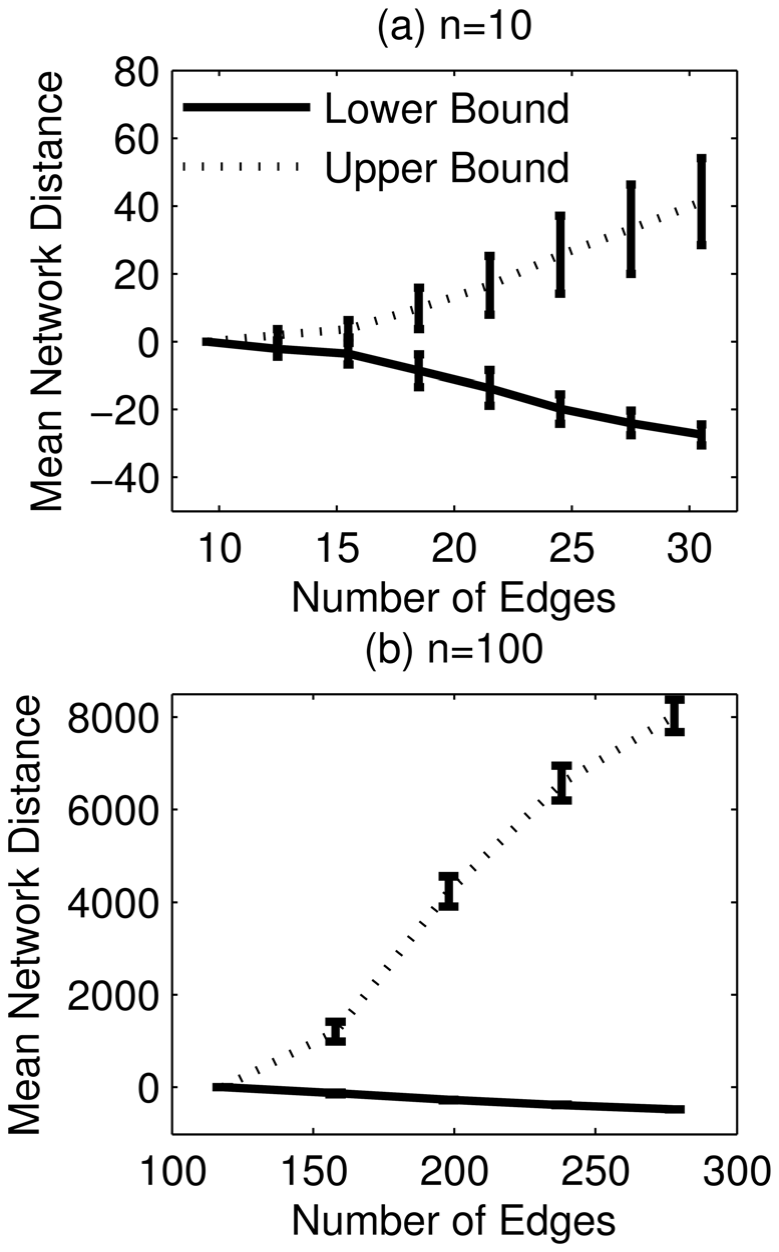
Ensemble inference and inferability of 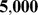 random scale-free networks of order (a) 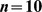 and (b) 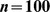 genes. The mean network distances of the lower and upper bounds from 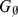 are shown as a function of network size (i.e. number of edges). The error bars indicate the standard deviations.

We used the error-free implementation of TRaCE to construct the ensemble lower and upper bounds for each of the random scale-free GRNs. Fig. 6 shows the mean network distances of the bounds as a function of network size. Similar to the random GRNs, most (79% for 10 gene networks and 75% for 100 gene networks) scale-free GRNs were not inferable from single and double-gene KO experiments, as the ensemble lower and upper bound did not meet for the majority of the networks. The mean network distance of the lower and upper bounds again increased with network size. However, the inference of scale-free GRNs from the accessibility matrices 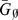 and 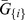
's appeared to be more difficult than that of random GRNs, as suggested by the larger distances between the lower and upper bounds for scale-free GRNs than for random GRNs of the same size.

#### *E. coli* and *S. cerevisiae* GRNs

**Figure 7:**
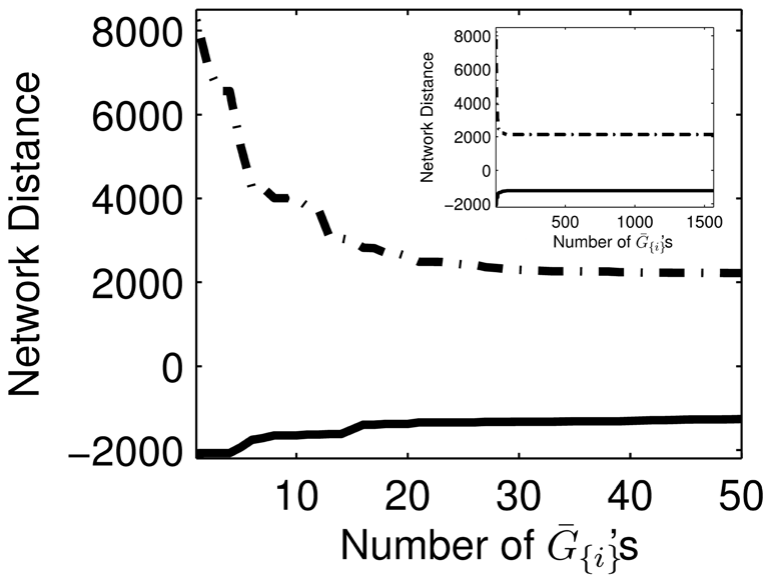
Ensemble inference of *E. coli* GRN from error-free 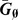 and the complete set of 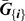
's. The plot shows the network distances of the lower and upper bounds from 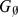 as a function of the number of 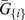
's for the 50 most informative 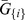
's, i.e. the top 50 highest number of testable edges. The incorporation of 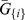
's and 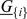
's was performed sequentially in decreasing number of testable edges. The inset shows the result for the complete set of 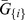
's.

**Figure 8:**
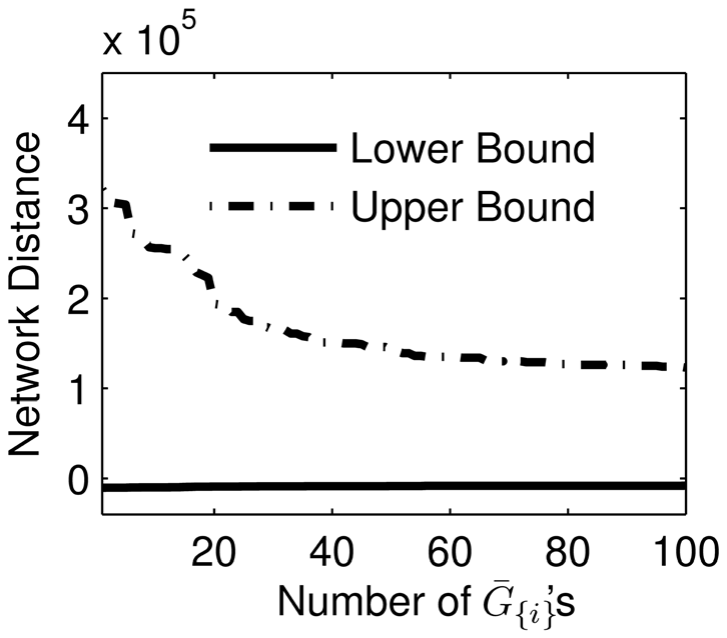
Ensemble inference of *S. cerevisieae* GRN from error-free 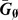 and the 100 most informative 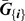
's based on the number of testable edges. The plot shows the network distances of the lower and upper bounds from 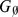 as a function of the number of 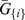
's. The incorporation of 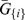
's and 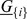
's was performed sequentially in decreasing number of testable edges.

Finally, we investigated the inferability of large, realistic GRNs of *E. coli* and *S. cerevisiae* available in GeneNetWeaver [24]. The *E. coli* GRN consists of 1565 genes and 3758 edges, while the yeast GRN comprise 4441 genes and 12873 edges. For *E. coli*, we generated the accessibility matrices of 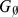 and all 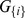
's. To reduce computational complexity, in the case of yeast, we used only the 100 most informative 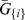
's based on the number of testable edges (i.e. the number of non-zero elements in the testability matrix 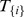 in Eq. (4)). The results are shown in Figs. 7 and 8. Not surprisingly, both *E. coli* and yeast GRNs could not be completely inferred from the above accessibility matrices. There was a diminishing return of information after about 25 and 50 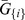
's for the inference of *E. coli* and yeast GRNs, respectively.

### Ensemble inference from errorneous accessibility matrices

We evaluated the performance of TRaCE with error correction using *E. coli* GRN and subnetworks, as well as yeast GRN. False positive errors were simulated by randomly adding edges to the accessibility matrices, while false negatives were simulated by randomly removing edges from the accessibility matrices. The performance of error correction in TRaCE was judged by the number of erroneous edges that remained in the bounds after correction for different FP and FN rates (abbreviated as FPR and FNR, respectively), defined with respect to the size of 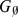.

#### *E. coli* GRNs

We first used TRaCE with error correction for the ensemble inference of 50 random subnetworks of *E. coli* GRN with 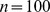 genes, generated using GeneNetWeaver [24]. The average number of edges was 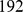. As in the above case study, we created the accessibility matrices of 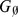 and every 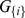. We subsequently contaminated these matrices with FP and FN errors at the specified rates without any preference. The accuracy of the lower and upper bounds constructed using TRaCE with and without error correction is summarized in Table 2.

**Table 2:** Ensemble inference of *E. coli* subnetworks (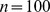 genes).

FPR	FNR	Before Correction		After Correction		
		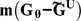	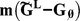	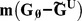	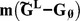	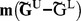
0.00	0.00	0	0	0	0	122.62
0.00	0.10	183.4	35.86	3.7	1.88	118.2
0.00	0.20	191.1	67.36	16.56	8.7	113.52
0.10	0.00	0	1412.42	0	0.24	150.4
0.10	0.10	183.02	1441.68	2.72	1.42	146.3
0.10	0.20	191	1460.82	12.06	2.1	146.74
0.20	0.00	0	2324.46	0	0.18	213.42
0.20	0.10	184.02	2354.16	2.72	0.46	197.28
0.20	0.20	191	2386.88	9.94	1.62	210.12

The reported values represent the averages over 50 subnetworks. FPR (FNR) is the ratio between the number of FP (FN) in the accessibility matrices and the number of edges in 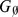. Let 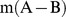 of any two digraphs 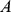 and 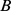 denote the number of edges in the set 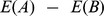.

As in many scenarios above, none of the subnetworks was inferable. FP errors could be very effectively eliminated by error correction. FNs errors expectedly led to missing edges from the upper bound, as indicated by the number of edges of 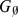 that did not appear in 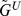 (see 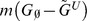 in Table 2). The error correction could not completely eliminate Type A errors, leading to erroneous edges that appeared in the lower bound 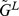 but did not belong to 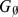 (see 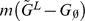 in Table 2). A combination of FP and FN errors were more easily corrected than FN errors alone. While FNs were more difficult to eliminate than FPs, correcting FP errors tended to produce larger network ensembles than FNs, indicating higher network uncertainty (see 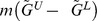 in Table 2). Nevertheless, even in the worst case (0% FP, 20% FN), roughly 90% of the errors in the lower and upper bounds could be removed by the error correction (compare 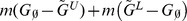 before and after correction).

For the inference of *E. coli* GRN, we generated erroneous accessibility matrices 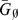 and the 100 most informative 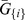
's corresponding to the top 100 highest numbers of testable edges. The performance of TRaCE with error correction for different FP and FN rates is summarized in Table 3. In addition, the structural Hamming distances of the lower and upper bounds before and after correction are reported in tables S1 and S2. As before, TRaCE with error correction could handle FPs more effectively than FNs, and a mixture of FP and FN errors in the accessibility matrices were more easily eliminated than FN alone. In the worst case (0% FP, 20% FN), more than 95% of the errors were corrected. The size of the ensemble also depended strongly on the FP errors, and at 20% FP, the number of edges between the lower and upper bound reached three times the size of the full GRN.

**Table 3:** Ensemble inference of *E. coli* GRN.

FPR	FNR	Before Correction		After Correction		
		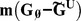	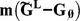	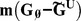	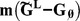	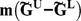
0.00	0.00	0	0	0	0	3351
0.00	0.10	3550	1029	25	46	3281
0.00	0.20	3746	1685	56	173	3092
0.10	0.00	0	34796	0	1	4636
0.10	0.10	3573	35566	14	1	4624
0.10	0.20	3746	35975	49	4	4556
0.20	0.00	0	62035	0	1	14933
0.20	0.10	3554	62632	12	7	14773
0.20	0.20	3741	63053	40	5	14392

FPR (FNR) is the ratio between the number of FP (FN) in the accessibility matrices and the number of edges in 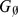. Let 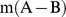 of any two digraphs 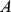 and 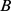 denote the number of edges in the set 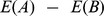.

#### *S. cerevisiae* GRN

For yeast GRN, we generated erroneous 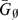 and the 100 most informative 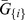
's. The results of TRaCE with error correction using these accessibility matrices are summarized in Table 4. The performance of TRaCE here was notably better than the inference of *E. coli* GRN. In all cases, TRaCE could rectify almost all erroneous edges. However, the correction came at a price of high uncertainty, where the difference between the lower and upper bounds exceeded 20 times the number of edges in 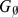. Despite such high uncertainty, the gap between the bounds represented only 1.3% of the total possible edges.

**Table 4:** Ensemble inference of *S. cerevisiae* GRN.

FPR	FNR	Before Correction		After Correction		
		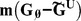	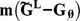	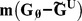	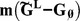	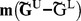
0.00	0.00	0	0	0	0	131595
0.00	0.10	4048	604	9	4	131879
0.00	0.20	6934	788	19	8	132155
0.10	0.00	0	121370	0	4	198624
0.10	0.10	4096	121563	8	4	198762
0.10	0.20	6879	121747	16	6	198883
0.20	0.00	0	227013	0	2	260484
0.20	0.10	4113	227087	4	3	260443
0.20	0.20	6909	227150	8	2	260313

Let 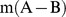 of any two digraphs 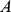 and 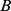 denote the number of edges in the set 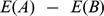.

### Ensemble inference from expression data

We further evaluated the performance of TRaCE using *in silico* noisy gene expression data generated using GeneNetWeaver [24]. We simulated steady-state gene expression data using the same settings as those in DREAM4 100-gene *in silico* network inference subchallenge. The simulated data are available at http://www.cabsel.ethz.ch/tools/trace or upon request. In the following case studies, we analyzed and converted the data into accessibility matrices following the procedure described in the Numerical Implementation section. Subsequently, we used the resulting accessibility matrices with TRaCE to produce the ensemble lower and upper bounds. For the purpose of comparison with existing network inference methods, we also ranked the gene regulatory interactions according to their confidence scores. In particular, we compared the rankings with those from top performing inference methods in DREAM4, namely the downranking method by Pinna et al. [27], GENIE3 [28] and TIGRESS [29]. As mentioned earlier, the downranking method follows a two-phase procedure in ranking edges similar to our implementation, but the method only down-ranks feed-forward edges. On the other hand, GENIE3 uses a machine learning strategy called random forest, and TIGRESS is a regression-based method. For each method, we calculated the area under receiver operating characteristics (AUROC) and precision-recall (AUPR) using a redefined confusion matrix, in which methods were not penalized for any error within the set of non-inferable edges. We define non-inferable edges as edges belonging to the upper bound that are missing from the lower bound (i.e. all edges in the set 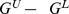), which are determined from error-free accessibility matrices of the gold standard GRNs. More details of the calculation of the AUROC and AUPR can be found in a recent publication [30].

#### DREAM4 *in silico* network inference 100-gene subchallenge

We first simulated 5 replicates of steady state gene expression data associated with the complete single-gene KO experiments. We then used the data to construct the accessibility matrices of the gold standard GRNs. In this case, the upper bound of the ensemble was simply given by the accessibility matrix, and the lower bound was the ConTREx of the upper bound. Table 5 shows the FPR and FNR in the accessibility matrices, the errors in the lower and upper bounds (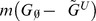 and 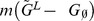), and the size of the ensemble (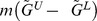) constructed using TRaCE. We noted that the majority (90%) of FN errors in the accessibility matrices were associated with fan-in motifs, in which a gene was regulated by several genes. In such a case, the effect of knocking-out one of the regulator genes could be compensated by the others and thus, the KO experiment did not show any significant differential expression of the downstream genes. As FNs affected the accessibility matrices, the errors in the upper bound 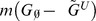 were higher than those in the lower bound 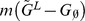.

**Table 5:** Ensemble inference of DREAM4 100-gene gold standard networks: single-gene KO dataset.

Network	FPR	FNR	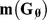	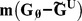	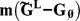	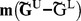
1	0	2.28	176	44	9	136
2	0	1.96	249	122	6	123
3	0	11.04	195	86	21	144
4	0	9.69	211	91	7	208
5	0	3.47	193	108	12	237

FPR (FNR) is the ratio between the number of FP (FN) in the accessibility matrices and the number of edges in 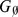. Let 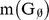 denote the number of edges in 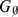, and 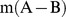 of any two digraphs 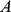 and 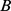 denote the number of edges in the set 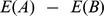.

**Figure 9:**
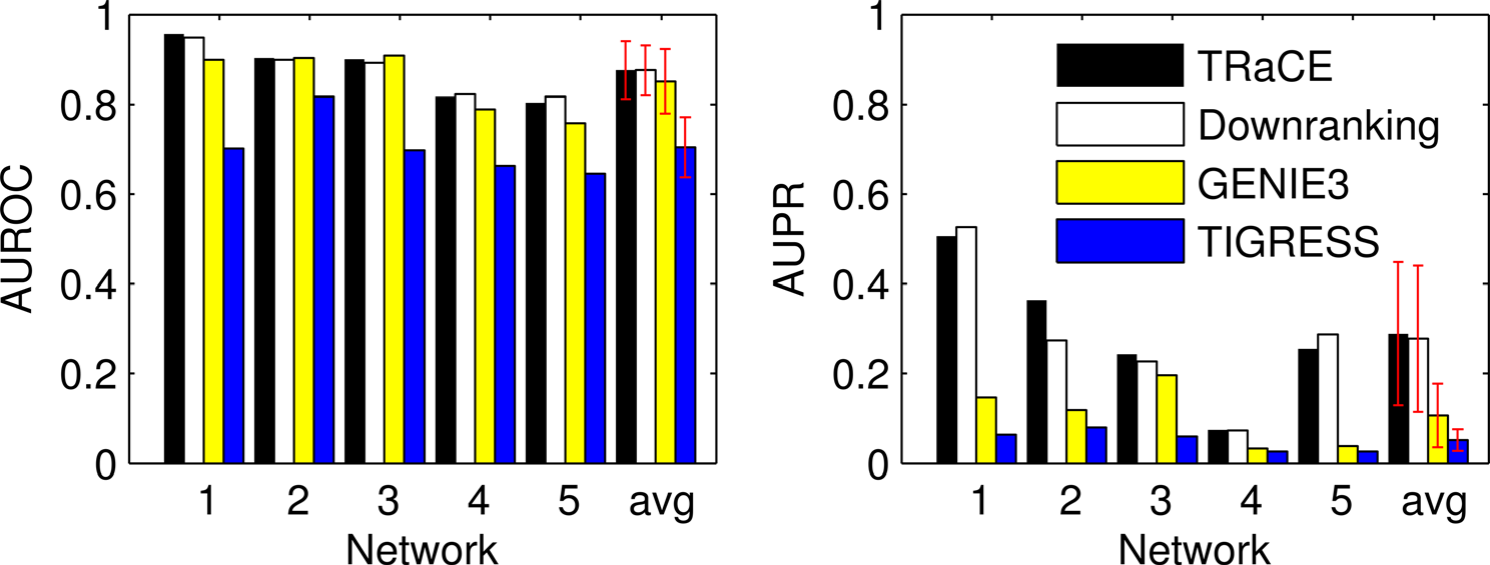
Comparison of TRaCE and top performing methods in DREAM4 100-gene network inference subchallenge: single-gene KO dataset. The error bars represent the standard deviations. Based on the AUROC values, TRaCE performed as well as the downranking method (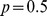) and GENIE3 (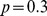), but better than TIGRESS (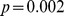). Based on the AUPR values, TRaCE performed as well as the downranking method (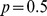), but better than GENIE3 (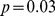) and TIGRESS (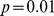). The statistical significance was evaluated using two sample t-test.

Subsequently, we created a ranked list of gene regulatory predictions and compared the list against those produced by the downranking method, GENIE3 and TIGRESS. Fig. 9 provides the comparison of AUROC and AUPR of the four methods. The comparison showed that TRaCE and the downranking method outperformed GENIE3 and TIGRESS, especially considering the AUPR values. Here, TRaCE performed as well as the downranking method, which was the best overall performer in DREAM 4 100-gene network inference subchallenge [27].

#### Ensemble inference from single- and double-gene Kos

We further simulated 5 replicates of steady state gene expression data for the complete set of single- and double-gene KO experiments using the gold standard GRNs of DREAM4 100-gene subchallenge. We processed the data to obtain the accessibility matrices of 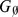 and all 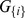
's. We subsequently applied TRaCE with error correction to the accessibility matrices to obtain the ensemble lower and upper bounds, which are summarized in Table 6. The average FPR and FNR were similar to the single-gene KO data since both datasets had the same number of replicates. Again, the majority (80%) of errors in the accessibility matrices were associated with fan-in motifs. By comparing Tables 5 and 6, the errors in the lower bounds improved slightly in comparison with those from only single-gene KO dataset (compare 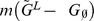 values). However, the errors in the upper bounds increased due to the accumulation of FN errors from fan-in motifs (compare 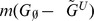 values). Nevertheless, the additional data from double-gene KO experiments led to lower network uncertainties (compare 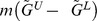 values).

**Table 6:** Ensemble inference of DREAM4 100-gene gold standard networks: single- and double-gene KO dataset.

Network	FPR	FNR	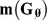	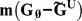	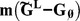	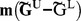
1	0.02	2.21	176	50	5	27
2	0.01	1.91	249	137	5	37
3	0	10.43	195	103	15	30
4	0	9.31	211	118	8	35
5	0.01	3.40	193	131	12	21

FPR (FNR) is the average ratio between the number of FP (FN) in the accessibility matrices and the number of edges in 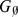. Let 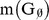 denote the number of edges in 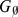, and 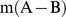 of any two digraphs 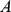 and 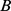 denote the number of edges in the set 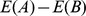.

**Figure 10:**
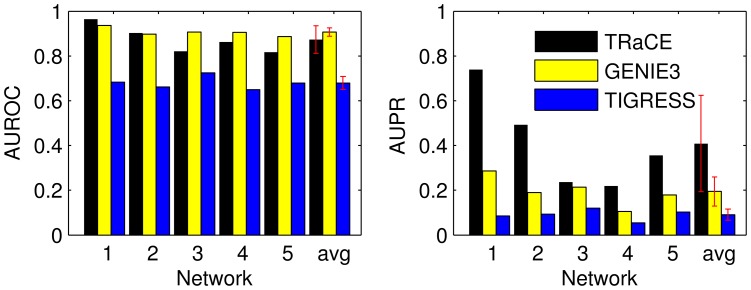
Comparison of TRaCE and top performing methods in DREAM4 100-gene network inference subchallenge: single- and double-gene KO dataset. The error bars represent the standard deviations. Based on the AUROC values, TRaCE performed as well as GENIE3 (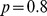) and better than TIGRESS (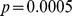). Similarly, based on the AUPR values, TRaCE performed better than GENIE3 (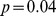) and TIGRESS (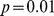). The statistical significance was evaluated using two sample t-test.

For each gold standard GRN, we also generated a ranked list of edges based on the confidence scores and compared the list with those using GENIE3 and TIGRESS. The downranking method could not be applied to double-gene KO data and was left out from the comparison. The AUROC and AUPR of the three methods are compared in Fig. 10. The AUPR of TRaCE was higher than GENIE3 and TIGRESS. Meanwhile, the AUROC values were generally high for all three methods, with TIGRESS having the lowest value. In comparison to single-gene KO data, the inclusion of double-gene KO data led to no change in the average AUROC (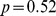, two sample t-test) and an increase in AUPR (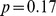, two sample t-test). Finally, as shown in Tables 7 and 8, the AUROC and AUPR values of TRaCE were insensitive to 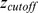 and 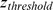 used in the construction of the accessibility matrices, and the threshold value in the preprocessing of 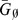. Because of the trade-off between FPs and FNs when using different 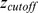 and 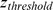, the maximum of AUROC and AUPR corresponded to intermediate values within the selected ranges of 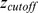 and 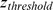.

**Table 7:** Effects of 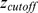 and 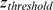 on AUROC and AUPR of TRaCE in DREAM4 100-gene subchallenge: single gene KO data.

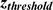	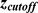	AUROC	AUPR
1.5	2	0.871 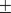 0.0616	0.3085 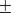 0.1416
1.5	3	0.8723 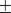 0.0673	0.3209 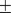 0.1534
2	2	0.8756 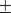 0.0595	0.2922 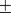 0.154
2	3	**0.8758** 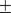 **0.0642**	**0.2889** 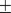 **0.1596**
2.5	3	0.8758 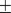 0.0642	0.2438 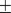 0.1545

The AUROC and AUPR values are the average 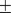 standard devation over 5 gold standard networks. The values used in the comparison with existing methods are highlighted in bold.

**Table 8:** Effects of 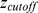, 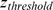 and the preprocessing threshold of 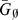 on AUROC and AUPR of TRaCE in DREAM4 100-gene subchallenge: single- and double-gene KO data.

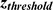	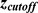	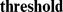	AUROC	AUPR
1.5	2	0.5	0.8723 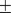 0.0595	0.4327 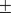 0.1931
		0.65	0.8718 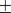 0.0588	0.4197 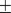 0.1959
		0.8	0.8724 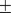 0.0600	0.4184 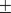 0.2022
1.5	3	0.5	0.8728 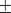 0.0596	0.4204 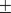 0.1986
		0.65	0.8733 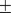 0.0610	0.4212 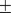 0.2059
		0.8	0.8735 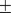 0.0617	0.4201 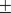 0.2100
2	2	0.5	0.8726 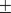 0.0603	0.4177 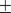 0.2158
		0.65	0.8727 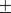 0.0607	0.4167 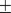 0.2170
		0.8	0.8728 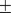 0.0608	0.4091 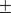 0.2139
2	3	0.5	0.8735 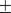 0.0618	0.4170 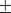 0.2160
		0.65	**0.8734** 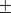 **0.0620**	**0.4086** 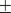 **0.2149**
		0.8	0.8698 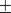 0.0604	0.4045 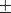 0.2108
2.5	3	0.5	0.8695 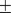 0.0599	0.3893 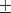 0.2054
		0.65	0.8696 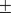 0.0598	0.3837 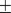 0.1975
		0.8	0.8695 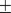 0.0600	0.3764 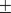 0.2023

The AUROC and AUPR values are the average 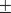 standard devation over 5 gold standard GRNs. The values used in the comparison with existing methods are highlighted in bold.

#### *E. coli* and Yeast GRNs

Finally, we simulated the complete set of single-gene KO experiments for *E. coli* and yeast. For each organism, we generated 10 replicates of steady state gene expression data. We performed differential expression analysis using the Z-score transformation and obtained the accessibility matrices using either 5 or 10 replicates. We then applied TRaCE with error correction to construct the ensemble lower and upper bounds, and created ranked lists of edges as done earlier. The errors in the accessibility matrices and in the bounds are reported in Table 9, along with the AUROC and AUPR values. Here, FPR in the accessibility matrices decreased with increasing number of replicates, but FNR did not change with the number of replicates. The errors in the upper bounds changed little with increasing technical replicates from 5 to 10, but those in the lower bounds dropped considerably. The sizes of the ensemble also decreased with increasing replicates. Finally, the AUPR values improved slightly with higher replicates, while the AUROC values were insensitive with respect to the number of replicates.

**Table 9:** Ensemble inference of *E. coli* and Yeast GRNs from single-gene KO data.

Network	Replicates	FPR	FNR	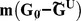	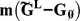	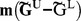	AUROC	AUPR
*E. coli*	5	0.117	2.61	1611	687	1724	0.8601	0.4836
*E. coli*	10	0.005	2.61	1612	297	1673	0.8639	0.5192
Yeast	5	0.582	24.97	8101	9159	7907	0.7699	0.2464
Yeast	10	0.141	24.98	8098	3753	7200	0.7569	0.2768

FPR (FNR) is the ratio between the number of FP (FN) in the accessibility matrices and the number of edges in 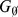. Let 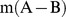 of any two digraphs 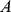 and 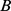 denote the number of edges in the set 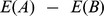.

## Discussion

The inference of GRNs from data of gene perturbation experiments is an important but unsolved problem. The difficulty stems from the underdetermined nature of such an inference [7], [9], as the data do not contain the necessary information to establish the complete causal interactions among the genes. Consequently, there exist many indistinguishable solutions. We have developed TRaCE with this consequence in mind by employing an ensemble inference strategy. Specifically, we have taken into consideration the fundamental limitation in using steady-state expression data of gene KO experiments for establishing direct causal relationships among genes. In TRaCE, we first transform the expression data into accessibility relationships (matrices) among genes. The novel contribution of TRaCE is an algorithm for the construction of lower and upper bounds of network ensemble, where each member of the ensemble satisfies the accessibility matrices. Edges of the upper bound that do not appear in the lower bound are considered non-inferable, as the existence of such edges can not be verified. Here, the size of the ensemble provides a metric of uncertainty in the network inference problem, with which the GRN inferability can be rigorously assessed. The GRN is inferable when the lower and upper bounds coincide (i.e. the ensemble only contains one network). Thus, in TRaCE, the inference and inferability analysis are accomplished simultaneously.

In the case studies, we have demonstrated the use of TRaCE for analyzing the inferability of GRNs. With the exception of networks of low order and small size, the majority of the GRNs were not inferable even when using error-free accessibility matrices. As we have used sparse networks in the case studies, the lower bound of the ensemble was a better estimate of the GRN than the upper bound. Finally, the majority of double-gene KO experiments were non-informative as the reduction in the size of the ensemble diminished after only a small number of 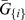
's. The observation above suggests that experimental design contributes significantly to the underdetermined nature of the typical GRN inference. In this regard, the lower and upper bounds of the ensemble could be used for optimizing the gene perturbation experiments, for example by finding the KO experiment that provides the maximum reduction in the difference between the lower and upper bounds. A strategy for optimal design of experiments using ensemble inference will be presented in a future publication.

We have also used the ensemble lower and upper bounds in conjunction with the z-scores to produce a ranked list of gene regulatory predictions. In comparison with the top methods of DREAM4 network inference challenge, TRaCE could match the performance of the downranking method, the best overall performer in the 100-gene subchallenge. For single-gene KO dataset, TRaCE and the downranking method differed only for edges that were involved in cycles of more than 2 nodes. However, the two methods were fundamentally different, as TRaCE was developed for ensemble inference. Furthermore, the downranking method was created for single-gene KO experiments. Meanwhile, TRaCE significantly outperformed GENIE3 and TIGRESS when using single- and double-gene KO data. We note that GENIE3 and TIGRESS were also among the best performers in DREAM5 network inference challenge [10].

As expected, data noise negatively influenced the GRN inference and increased the uncertainty in the GRN inference. In the case studies, random errors in the accessibility matrices expectedly led to a larger ensemble. While the error correction in TRaCE was able to eliminate the majority of errors, some of the errors remained in the bounds. In particular, FN errors were harder to correct than FPs. The reason was that more Type A errors originating from FNs passed through the correction than those originating from FPs (see Fig. S3 and text S4). Meanwhile, FP errors could cancel out some Type A errors associated with FNs at the cost of increased uncertainty (see Fig. S4 and text S4).

The application of TRaCE to simulated noisy gene expression data indicated that the majority of errors in the accessibility matrices were due to FNs associated with fan-in motifs in the GRN. In such motifs, the effects of knocking-out one regulator gene could be compensated by other regulator(s), and differential expression analysis could only reveal the dominant regulator(s) of a gene. Note that such a problem could not be improved by increasing technical replicates (see Table 9). Meanwhile, errors associated with fan-in motifs usually lead to type A errors where the affected edges are absent from the lower and upper bounds. However, if the available experiments permit the construction of 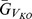 in which 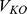 includes the dominant regulator(s) of a fan-in motif, then the related missing edge(s) may appear in 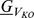, leading to a detectable and correctable type C error. We expect the issue above would improve when using more sensitive measurements of gene expression, for example RNAseq.

## Conclusion

Inferring gene regulatory networks from DNA microarray data is an unsolved problem. Community-wide assessments of inference methods have shown that distinguishing direct and indirect regulatory interactions among genes is a common Achilles' heel of existing algorithms. In this study, we have adopted an ensemble inference strategy and develop new framework and algorithms for the creation of an ensemble of networks. Here, the ensemble represents the uncertainty associated with differentiating direct and indirect regulations using steady-state gene expression data of gene KO experiments. In particular, TRaCE produces the lower and upper bound of the ensemble. Using the bounds of the ensemble, a ranked list of gene regulatory predictions can also be generated. The case studies demonstrate that except for networks with few edges, most GRNs can not be fully inferred even when error-free data from complete single- and double-gene knock out experiments are available. In comparison with top performing methods of DREAM4 *in silico* network inference challenge, TRaCE performed equally well with the downranking method, the best overall performer in the challenge. However, the downranking method is not designed to handle data from multi-gene KO experiments. Meanwhile, TRaCE outperformed GENIE3 and TIGRESS when using single- and double-gene KO data. Nevertheless, the uncertainty in GRN inference is still significant, and systematic KOs of genes are often suboptimal as only a small fraction of the experiments are informative. We therefore hope that the shift of paradigm to ensemble inference will trigger further developments of methodologies for inferring and analyzing GRNs, in which network inference uncertainty is explicitly taken into consideration.

## Supporting Information

Figure S1
**An example of ConTREx of a simple GRN.** The directed edges indicated by blue arrows in 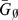 are in the set of indirect regulations. The edges between A and B are retained, because the cycle contains only two nodes. If the cycle had contained more than two nodes, all edges among the nodes would have been removed.(TIFF)

Figure S2
**Example of an ensemble involving GRN with cycle.** Consider a GRN consisting of genes A, B and C, all of which are involved in a directed cycle. Further, let us assume that the edge 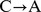 belongs to the lower bound and 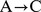 is not in the the upper bound. In this case, the ensemble comprises the graphs shown in (a)-(d).(TIFF)

Figure S3
**Type A errors due to an FN.** (a) 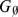; (b) 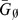; (c) 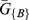; (d) 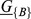; (e) 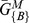 with an FN at 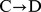. In this case, 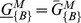. (f) 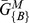 with an FN at 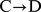 and an FP at 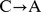 (g) ConTREx reduction of (f). (h) 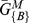 with an FN at 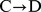 and an FP at 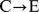 (i) ConTREx reduction of (h). See text S4 for details.(TIFF)

Figure S4
**Type A error due to an FP.** 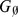 and 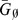 are shown in Fig. S3 (a) and (b), respectively. (a) 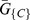. In this case, 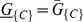. (b) 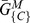
with an FP at 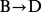. Here, 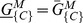.(TIFF)

Table S1
**Performance of TRaCE on inference of** ***E. coli*** **subnetworks (** 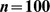 **genes).** The reported values represent the average over 50 subnetworks. Let 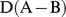 of any two digraphs 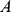 and 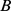 denote the structural Hamming distance (SHD) between them. The SHD is defined as the number of edges which differ or have opposite orientation between two networks [31].(PDF)

Table S2
**Performance of TRaCE on inference of** ***E. coli*** **GRN.** Let 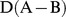 of any two digraphs 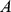 and 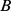 denote the structural Hamming distance between them.(PDF)

Text S1
**Preprocessing of** 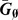 **.**
(PDF)

Text S2 **Filter Algorithms.**
(PDF)

Text S3 **Consistency check.**
(PDF)

Text S4 **Type A errors due to FN.**
(PDF)
