# Supplementary figures and images for: Ensemble Inference and Inferability of Gene Regulatory Networks
Source: PLoS One. 2014 Aug 5;9(8):e103812. doi: 10.1371/journal.pone.0103812 (PMC4122380; doi:10.1371/journal.pone.0103812)

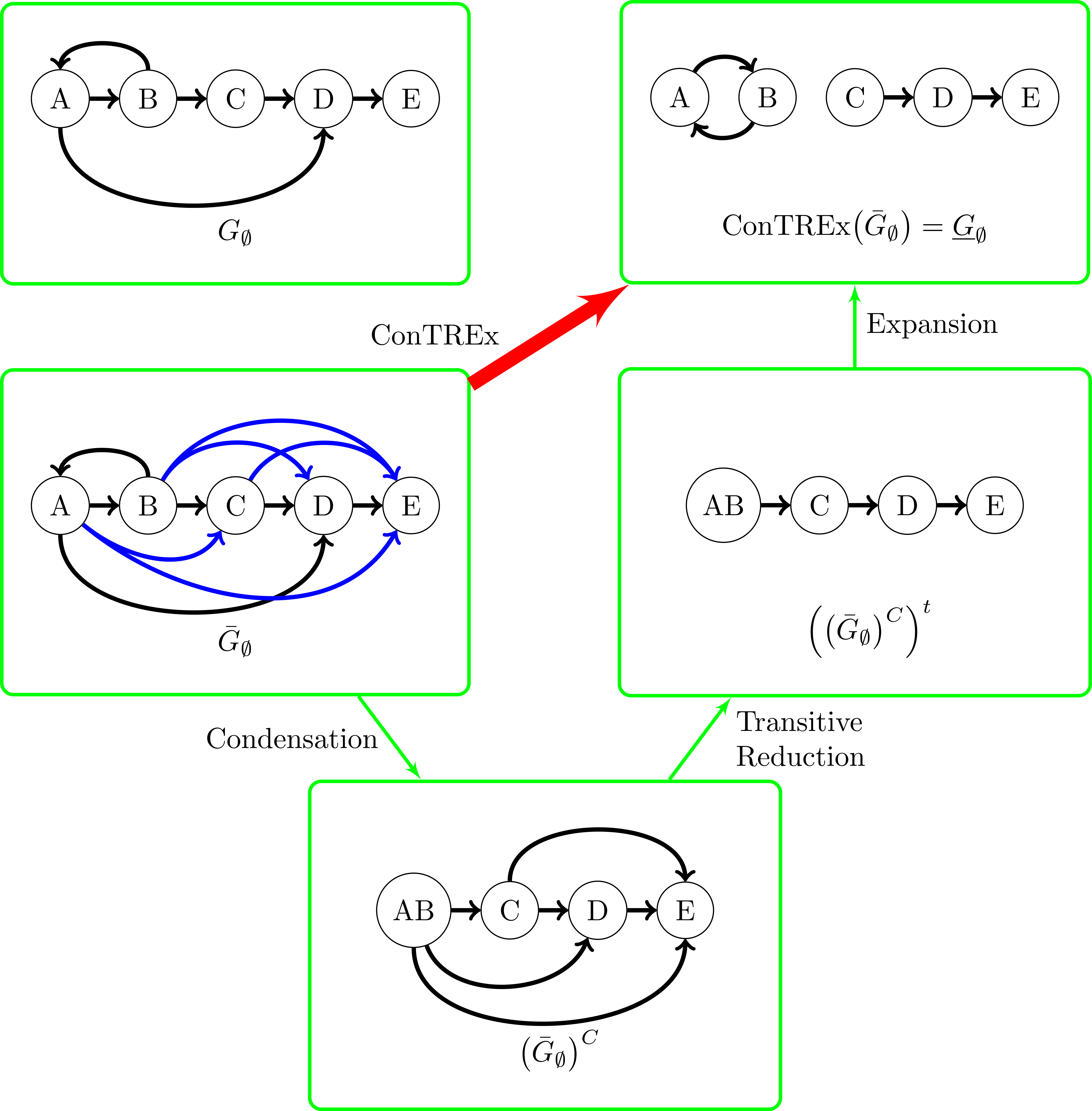

Supplement: Figure S1 — An example of ConTREx of a simple GRN. The directed edges indicated by blue arrows in are in the set of indirect regulations. The edges between A and B are retained, because the cycle contains only two nodes. If the cycle had contained more than two nodes, all edges among the nodes would have been removed. (TIFF) [file pone.0103812.s001.tiff]

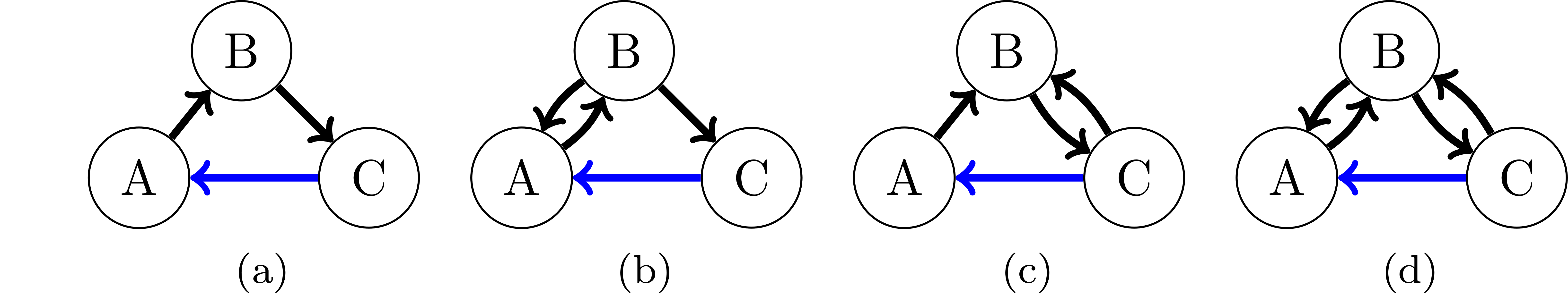

Supplement: Figure S2 — Example of an ensemble involving GRN with cycle. Consider a GRN consisting of genes A, B and C, all of which are involved in a directed cycle. Further, let us assume that the edge belongs to the lower bound and is not in the the upper bound. In this case, the ensemble comprises the graphs shown in (a)-(d). (TIFF) [file pone.0103812.s002.tiff]

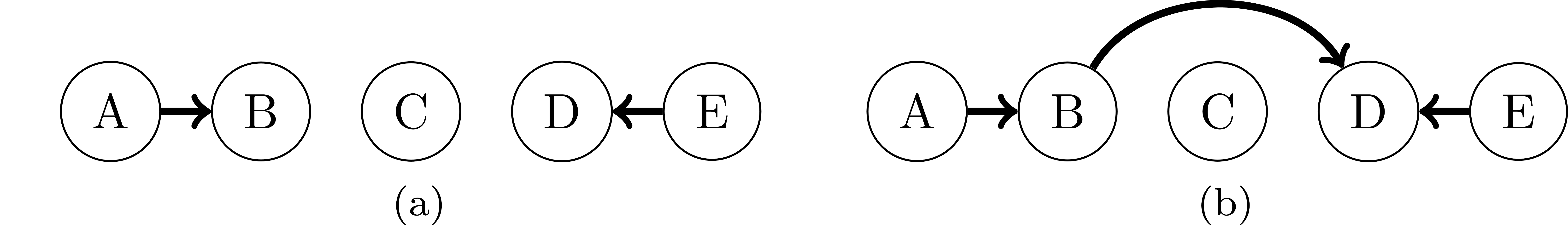

Supplement: Figure S4 — Type A error due to an FP. and are shown in Fig. S3 (a) and (b), respectively. (a) . In this case, . (b) with an FP at . Here, . (TIFF) [file pone.0103812.s004.tiff]
