# Supplementary material for: Ensemble Inference and Inferability of Gene Regulatory Networks
Source: PLoS One. 2014 Aug 5;9(8):e103812. doi: 10.1371/journal.pone.0103812 (PMC4122380; doi:10.1371/journal.pone.0103812)
Supplement: Table S1 — Performance of TRaCE on inference of E. coli subnetworks ( genes). The reported values represent the average over 50 subnetworks. Let of any two digraphs and denote the structural Hamming distance (SHD) between them. The SHD is defined as the number of edges which differ or have opposite orientation between two networks [31]. (PDF) [file pone.0103812.s005.pdf]

FPR	FNR	Before Correction		After Correction		
		$\mathtt{D}(\mathtt{G}_\emptyset, \tilde{\mathtt{G}}^{\mathtt{U}})$	$\mathtt{D}(\tilde{\mathtt{G}}^{\mathtt{L}}, \mathtt{G}_\emptyset)$	$\mathtt{D}(\mathtt{G}_\emptyset, \tilde{\mathtt{G}}^{\mathtt{U}})$	$\mathtt{D}(\tilde{\mathtt{G}}^{\mathtt{L}}, \mathtt{G}_\emptyset)$	$\mathtt{D}(\tilde{\mathtt{G}}^{\mathtt{U}}, \tilde{\mathtt{G}}^{\mathtt{L}})$
0.00	0.00	63.5	54.6	63.5	54.6	118.1
0.00	0.10	189.4	85.9	70.9	59.9	114.0
0.00	0.20	191.7	110.8	97.7	86.2	107.4
0.10	0.00	63.8	1490.5	81.1	75.1	155.5
0.10	0.10	188.5	1510	84.6	83.1	154.8
0.10	0.20	191.6	1525.9	101.3	93.2	151.0
0.20	0.00	63.8	2422.2	133.7	93.6	226.8
0.20	0.10	189.8	2440.0	124.4	93.8	208.6
0.20	0.20	191.5	2469.2	148.3	105.8	219.6

Table S1: Performance of TRaCE on inference of *E. coli* subnetworks ($n = 100$ genes). The reported values represent the average over 50 subnetworks. Let $\mathtt{D}(\mathtt{A} - \mathtt{B})$ of any two digraphs A and B denote the structural Hamming distance between them.
