# Supplementary material for: Ensemble Inference and Inferability of Gene Regulatory Networks
Source: PLoS One. 2014 Aug 5;9(8):e103812. doi: 10.1371/journal.pone.0103812 (PMC4122380; doi:10.1371/journal.pone.0103812)
Supplement: Table S2 — Performance of TRaCE on inference of E. coli GRN. Let of any two digraphs and denote the structural Hamming distance between them. (PDF) [file pone.0103812.s006.pdf]

FPR	FNR	Before Correction		After Correction		
		$\mathtt{D}(\mathtt{G}_{\emptyset}, \tilde{\mathtt{G}}^{\mathtt{U}})$	$\mathtt{D}(\tilde{\mathtt{G}}^{\mathtt{L}}, \mathtt{G}_{\emptyset})$	$\mathtt{D}(\mathtt{G}_{\emptyset}, \tilde{\mathtt{G}}^{\mathtt{U}})$	$\mathtt{D}(\tilde{\mathtt{G}}^{\mathtt{L}}, \mathtt{G}_{\emptyset})$	$\mathtt{D}(\tilde{\mathtt{G}}^{\mathtt{U}}, \tilde{\mathtt{G}}^{\mathtt{L}})$
0.00	0.00	2132	1193	2132	1193	3325
0.00	0.10	3661	2160	2185	1285	3254
0.00	0.20	3752	2671	2228	1529	3067
0.10	0.00	2134	36408	3021	1591	4609
0.10	0.10	3676	36922	3043	1599	4597
0.10	0.20	3749	37171	3103	1585	4529
0.20	0.00	2134	63975	13001	1910	14908
0.20	0.10	3680	64348	12887	1918	14748
0.20	0.20	3746	64701	12583	1919	14367

Table S2: Performance of TRaCE on inference of *E. coli* GRN. Let $\mathtt{D}(\mathtt{A}-\mathtt{B})$ of any two digraphs A and B denote the structural Hamming distance between them.
