# Supplementary material for: Ensemble Inference and Inferability of Gene Regulatory Networks
Source: PLoS One. 2014 Aug 5;9(8):e103812. doi: 10.1371/journal.pone.0103812 (PMC4122380; doi:10.1371/journal.pone.0103812)
Supplement: Text S1 — Preprocessing of . (PDF) [file pone.0103812.s007.pdf]

Preprocessing of $\bar{G}_{\emptyset}$

In general, accessibility matrices inferred from noisy measurements will have FP and FN errors. To correct errors in $\bar{G}_{\emptyset}$, we have made the assumption that errors rarely occur for the same edge among input accessibility matrices. Hence, an edge that appears in a majority of the input matrices (beyond a certain threshold) was added to the $\bar{G}_{\emptyset}$, or was otherwise removed from $\bar{G}_{\emptyset}$. This procedure is implemented as follows:

$$A = Acc\left(\bar{G}_{\emptyset}\right) + \sum_i Acc\left(\bar{G}_{\{i\}}\right)$$

$$Acc_{i,j}\left(\widetilde{\bar{G}_{\emptyset}}\right) = \begin{cases} 1 & A_{i,j} \geq \texttt{threshold} \\ 0 & \texttt{otherwise} \end{cases}$$

Here, $\left(\widetilde{\bar{G}_{\emptyset}}\right)$ denotes the corrected $\bar{G}_{\emptyset}$. In our experience, we could obtain good results with $\texttt{threshold} = 0.65 \times N_{acc}$, where N_{acc} is the number of input accessibility matrices. In general, the performance of TRaCE with error correction was not sensitivity for `threshold` values in the range between $0.5 \times N_{acc}$ and $0.8 \times N_{acc}$.
