# Supplementary material for: Ensemble Inference and Inferability of Gene Regulatory Networks
Source: PLoS One. 2014 Aug 5;9(8):e103812. doi: 10.1371/journal.pone.0103812 (PMC4122380; doi:10.1371/journal.pone.0103812)
Supplement: Text S2 — Filter Algorithms. (PDF) [file pone.0103812.s008.pdf]

## Filter Algorithms

### Filter for $\tilde{G}^L$

The filter algorithm below is designed to prevent the propagation of FP edges in any $\underline{G}_{\{i\}}$ to the lower bound $\tilde{G}^L$. The pseudocode for the union with filter $\dot{\bigcup}$ is given below:

$Adj\left(\tilde{G}^L\right) = Adj\left(\underline{\tilde{G}}_\emptyset\right)$

$A = Acc(\widetilde{\bar{G}_\emptyset})$

FOR every k

$A_{test} = \sum_{i \in V_{KO}^k} A_i \otimes A^i$

$Adj\left(\tilde{G}^L\right) = Adj\left(\tilde{G}^L\right) + A_{test} \odot Adj\left(\underline{G}_{V_{KO}^k}\right)$

END FOR

where $\otimes$ denotes the outer product and $\odot$ denotes the Hadamard multiplication (element wise multiplication). Note that the elements of the matrices are either 0 or 1, and the addition operation + denotes a Boolean sum.

### Filter for $\tilde{G}^U$

The following filter is created to avoid propagating FN edges in any $\bar{G}_{\{i\}}$ to $\tilde{G}^U$. The pseudocode for the intersection with filter $\dot{\bigcap}$ is given below:

$Adj\left(\tilde{G}^U\right) = Acc(\widetilde{\bar{G}_\emptyset})$

$A = Acc(\widetilde{\bar{G}_\emptyset})$

FOR every $k$

$A_{test} = \sum_{i \in V_{KO}^k} A_i \otimes A^i$

$Adj\left(\tilde{G}^U\right) = Adj\left(\tilde{G}^U\right) \odot A_{test} \odot Adj\left(\bar{G}_{V_{KO}^k}\right)$

END FOR
