# Supplementary material for: Ensemble Inference and Inferability of Gene Regulatory Networks
Source: PLoS One. 2014 Aug 5;9(8):e103812. doi: 10.1371/journal.pone.0103812 (PMC4122380; doi:10.1371/journal.pone.0103812)
Supplement: Text S3 — Consistency check. (PDF) [file pone.0103812.s009.pdf]

Consistency check

The set of inconsistent edges can be readily identified by the set difference $\tilde{G}^L - \tilde{G}^U$. An inconsistent edge arises due to conflicting evidence about the existence or non-existence of an edge. This means that for some input accessibility matrices, this particular edge is a member of both $\bar{G}$ and $\underline{G}$, while for other input matrices, the edge is absent from $\bar{G}$ and $\underline{G}$. To resolve inconsistent edges, we take a vote among input matrices that are in support for or against the existence of each of these edges. When an inconsistent edge receives a majority positive vote (supporting its existence), the upper bound $\tilde{G}^U$ is updated to include the edge. Otherwise, this edge is removed from $\tilde{G}^L$. In case of a tie vote, the edge is removed from $\tilde{G}^L$ and added to $\tilde{G}^U$ such that $\tilde{G}^L \subset \tilde{G}^U$. The pseudocode for the consistency check algorithm (CC) is given below:

```
FOR each inconsistent edge (i, j)
  T_vote = 0
  F_vote = 0
  FOR every k
    IF Adj(Ḡ_{V^k_KO})_{i,j} = 1 AND Adj(G̲_{V^k_KO})_{i,j} = 1 THEN T_vote = T_vote + 1
    ELSEIF Adj(Ḡ_{V^k_KO})_{i,j} = 0 AND Adj(G̲_{V^k_KO})_{i,j} = 0 THEN F_vote = F_vote + 1
    END IF
  END FOR
  IF T_vote > F_vote THEN Adj(G̃^U)_{i,j} = 1
  ELSEIF T_vote < F_vote THEN Adj(G̃^L)_{i,j} = 0
  ELSE Adj(G̃^U)_{i,j} = 1 AND Adj(G̃^L)_{i,j} = 0
  END IF
END FOR
```
