# Supplementary material for: Ensemble Inference and Inferability of Gene Regulatory Networks
Source: PLoS One. 2014 Aug 5;9(8):e103812. doi: 10.1371/journal.pone.0103812 (PMC4122380; doi:10.1371/journal.pone.0103812)
Supplement: Text S4 — Type A errors due to FN. (PDF) [file pone.0103812.s010.pdf]

Type A errors due to FN

Some Type A errors which originate due to FN errors in the $\bar{G}_{\{i\}}$'s are transmitted to the lower and upper bounds. While it is intuitive that FP errors in any $\bar{G}_{\{i\}}$'s could lead to erroneous edges in the lower bound, FNs can also give rise to the same kind of errors. An example of this scenario is illustrated in Fig. S3 (a)-(e). Here, we focus on the edge A $\rightarrow$ D (blue arrow). Based on $\bar{G}_{\emptyset}$ in Fig. S3 (b), the edge A $\rightarrow$ D is deemed testable when incorporating $\bar{G}_{\{B\}}$ and its ConTREx, $\underline{G}_{\{B\}}$ (see Fig. S3 (c)-(d)). Let us consider an FN error associated with a edge C $\rightarrow$ D missing in the measured $\bar{G}_{\{B\}}$, denoted by $\bar{G}^{M}_{\{B\}}$. Because of this error, A $\rightarrow$ D edge which should have been removed in the ConTREx, now erroneously appears in $\underline{G}^{M}_{\{B\}}$, as shown in Fig. S3 (e). Since A $\rightarrow$ D is a testable edge, the A $\rightarrow$ D error in $\underline{G}^{M}_{\{B\}}$will pass through the filter. In general, an erroneous edge between two arbitrary nodes j and k can appear in the output of ConTREx due to an FN error, when this error breaks all indirect path(s) between j and k. In addition, if such an error occurs in the input matrix $\bar{G}_{\{i\}}$ with gene i on any path between j and k , the erroneous edge will be deemed testable and therefore will pass through the filter algorithm.

There are two circumstances when an FP error can compensate Type A errors originated from an FN in the above example. The first is when the FP errors lead to the creation of cycle(s) to which the Type A error above is incident. For the example above, an FP involving C $\rightarrow$ A in $\bar{G}^{M}_{\{B\}}$ will form a directed cycle between nodes A and C (see Fig. S3 (f)), the ConTREx of this input is shown in Fig. S3 (g). In this case, the edge A $\rightarrow$ D is removed as this edge is incident to a cycle. Alternatively, an FP can create a new indirect path between the nodes associated with the Type A error edge above. In the simple example, when the FN in $\bar{G}^{M}_{\{B\}}$ affecting C $\rightarrow$ D edge is accompanied with an FP of the edge C $\rightarrow$ E (see Fig. S3 (h)), the edge A $\rightarrow$ D will be pruned during ConTREx and thus will not appear in $\underline{G}^{M}_{\{B\}}$. Finally, the difference between Type A error in $\tilde{G}^{L}$ induced by FP and FN is that the FP error needs to occur at the affected edge (see Fig. S4), while the number of scenarios for which this error occurs by way of FN is more numerous. For this reason, there are more Type A errors due to FN that escape the correction algorithm than those due to FP.
